# Efficacy and safety of stem cell transplantation for multiple sclerosis: a systematic review and meta-analysis of randomized controlled trials

**DOI:** 10.1038/s41598-024-62726-4

**Published:** 2024-05-31

**Authors:** Asmaa Ahmed Nawar, Aml Mostafa Farid, Rim Wally, Engy K. Tharwat, Ahmed Sameh, Yomna Elkaramany, Moamen Mostafa Asla, Walaa A. Kamel

**Affiliations:** 1 Faculty of Medicine, Zagazig University, Zagazig, Egypt; 2 Faculty of Dentistry, Suez Canal University, Ismailia, Egypt; 3 Bioinformatics Group, Centre for Informatics Science, School of Information Technology and Computer Science, Nile University, Giza, Egypt; 4 Biotechnology Department, Faculty of Science, Cairo University, Giza, Egypt; 5 Neurology Department, Faculty of Medicine, Beni-Suef University, Beni-Suef, Egypt

**Keywords:** Multiple sclerosis, Stem-cell research, Neurology

## Abstract

Multiple sclerosis (MS) is a common autoimmune neurological disease affecting patients’ motor, sensory, and visual performance. Stem Cell Transplantation (SCT) is a medical intervention where a patient is infused with healthy stem cells with the purpose of resetting their immune system. SCT shows remyelinating and immunomodulatory functions in MS patients, representing a potential therapeutic option. We conducted this systematic review and meta-analysis that included randomized control trials (RCTs) of SCT in MS patients to investigate its clinical efficacy and safety, excluding observational and non-English studies. After systematically searching PubMed, Web of Science, Scopus, and Cochrane Library until January 7, 2024, nine RCTs, including 422 patients, were eligible. We assessed the risk of bias (ROB) in these RCTs using Cochrane ROB Tool 1. Data were synthesized using Review Manager version 5.4 and OpenMeta Analyst software. We also conducted subgroup and sensitivity analyses. SCT significantly improved patients expanded disability status scale after 2 months (N = 39, MD =  − 0.57, 95% CI [− 1.08, − 0.06], *p* = 0.03). SCT also reduced brain lesion volume (N = 136, MD = − 7.05, 95% CI [− 10.69, − 3.4], *p* = 0.0002). The effect on EDSS at 6 and 12 months, timed 25-foot walk (T25-FW), and brain lesions number was nonsignificant. Significant adverse events (AEs) included local reactions at MSCs infusion site (N = 25, RR = 2.55, 95% CI [1.08, 6.03], *p* = 0.034) and hematological disorders in patients received immunosuppression and autologous hematopoietic SCT (AHSCT) (N = 16, RR = 2.33, 95% CI [1.23, 4.39], *p* = 0.009). SCT can improve the disability of MS patients and reduce their brain lesion volume. The transplantation was generally safe and tolerated, with no mortality or significant serious AEs, except for infusion site reactions after mesenchymal SCT and hematological AEs after AHSCT. However, generalizing our results is limited by the sparse number of RCTs conducted on AHSCT. Our protocol was registered on PROSPERO with a registration number: CRD42022324141.

## Introduction

Multiple Sclerosis (MS) is a common autoimmune demyelinating neurological disease affecting more than 2.8 million patients worldwide^1^. It presents with different manifestations such as visual loss, weakness, sensory, and even sphincteric disturbances^2^. MS includes five main clinical courses: Primary Progressive Multiple Sclerosis (PPMS), Secondary Progressive Multiple Sclerosis (SPMS), Relapsing–Remitting Multiple Sclerosis (RRMS), Clinically Isolated Syndrome (CIS), and Radiologically Isolated Syndrome (RIS). These clinical phenotypes are assessed according to two descriptors: disease activity and progression. Disease activity is evidenced by clinical relapses or lesions activity on MRI, while disability progression is associated with increasing neurological dysfunction^3^.

Currently, approved medications for MS only aim to alleviate the symptoms or slow disease progression and reduce relapses through disease-modifying therapies (DMTs). Of these drugs, interferon beta (INF-β) and glatiramer acetate can reduce the relapse rate by one-third in relapsing MS (RMS; includes RRMS, CIS, and SPMS with relapses) through their immunomodulatory effect. Oral immunomodulators, including dimethyl-fumarate and teriflunomide, are also effective in reducing the disease activity in RMS^4,5^. Other DMTs can inhibit the migration of immune cells to the CNS, such as fingolimod which decreases the disease activity with considerable safety concerns, including bradycardia, liver injury, and infections. Siponimod is another novel cell migration inhibitor that decreases the relapse rate in SPMS by about 20% with the same adverse events as fingolimod^5^. For SPMS and worsening RMS, mitoxantrone induces immunosuppression but carries a high risk of cardiotoxicity and hematological malignancies^4^. Cladribine is another agent used for the relapsing forms of MS. Novel B-cell depleting drugs, including natalizumab, ocrelizumab, alemtuzumab, and ofatumumab can be used for RRMS and active SPMS^6,7^. However, patients with SPMS and PPMS have fewer options of medications with limited efficacy and safety issues. To date, finding an ultimate cure for MS is still an unmet need^8,9^.

Stem cell transplantation (SCT) has emerged as another treatment option for multiple sclerosis in addition to different autoimmune neurological diseases such as myasthenia gravis, Neuromyelitis optica, and systemic inflammatory diseases including rheumatoid arthritis and systemic lupus erythematosus^10,11^. SCT involves ablation of the patient's aberrant immune system and reconstitution of a new immune system derived after the infusion of healthy stem cells^12^. The European Group for Blood and Marrow Transplantation has recommended autologous hematopoietic SCT (AHSCT) for MS patients showing inflammatory disease activity, including RRMS patients not responding to the approved DMTs and SPMS patients with worsening disability^13^. Young and ambulatory MS patients are considered the optimal candidates for AHSCT^14^.

In multiple sclerosis, stem cells migrate into the brain lesions, contribute to regenerating the impaired myelin and induce tissue repair. This regenerative process is attributed to the ability of stem cells to differentiate into both neuronal and myelin-producing cells^15^. Stem cells also show immunomodulatory functions by inhibiting the autoimmune lymphocytes that attack the white matter of the brain, providing a neuroprotective potential as observed in preclinical and clinical studies^15,16^.

Clinical trials have detected promising clinical recovery and improvement of the quality of life of MS patients after SCT with minimal safety concerns^17–19^. However, these clinical trials show variations in the transplantation procedure, including the dose, the origin of the cells, and their route of administration. Stem cells can be found in embryonic tissue or in adult tissue, including hematopoietic stem cells (in the bone marrow or peripheral blood), mesenchymal stem cells (in bone marrow or adipose tissue), and neural stem cells in the brain^18,20^. Furthermore, autogenic stem cells are isolated from the patient who gets the transplantation, and allogenic stem cells are derived from a donor. Stem cells are usually infused via the intravenous route; however, the intrathecal or the intraventricular routes are expected to be more effective in MS^21^. These variations limited finding the transplantation approach that produces the optimal benefits for MS patients. We conducted this systematic review and meta-analysis of randomized control trials to assess the efficacy and safety of various SCT approaches in MS.

## Methods

Applying the Preferred Reporting Items for Systematic Review and Meta-Analyses (PRISMA) statement 2020 guidelines^22^, we proceeded as follows**:**

### Protocol and registration

Our protocol was registered on PROSPERO with a registration number: CRD42022324141.

### Eligibility criteria

We included studies satisfying the following PICOS (Population, Intervention, Comparator, Outcomes, and Study design) criteria in our meta-analysis: Population: patients with multiple sclerosis; Intervention: stem cells transplantation without restrictions on the dose or the source of stem cells; Comparator: control group received placebo or active treatment; Outcome: efficacy and/or safety of stem cell transplantation; Study design: randomized controlled trials (RCTs).

### Exclusion criteria

We excluded studies that didn’t meet the PICOS criteria, observational studies, animal or experimental trials, reviews, book chapters, conference abstracts, trial registries, and protocols.

### Information sources

**Figure 1:**
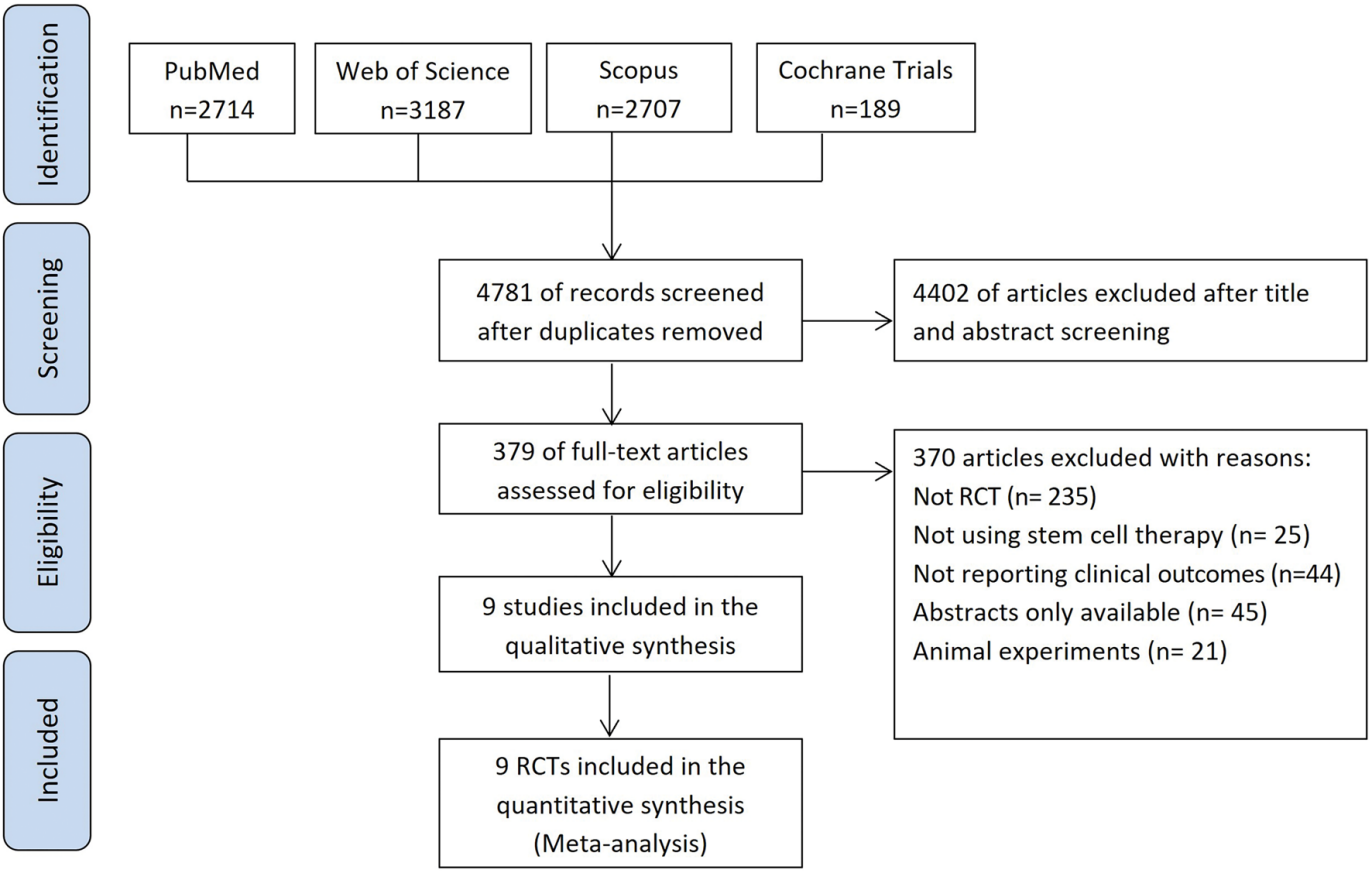
PRISMA Flow chart of the study selection process.

We searched four electronic databases: PubMed, Scopus, Web of Science (WOS), and Cochrane Central Register of Controlled Trials from inception to January 7, 2024. Figure 1 presents the flow diagram of studies selection.

### Search strategy

We used the following search terms: multiple sclerosis, disseminated sclerosis, stem cells, mesenchymal cells, and hematopoietic cells. The PubMed advanced search strategy was as follows: (((Multiple[Title/Abstract] OR Disseminated[Title/Abstract]) AND Sclerosis[Title/Abstract])) AND ((Stem[Title/Abstract] OR Hematopoietic[Title/Abstract] OR Progenitor[Title/Abstract] OR Mesenchymal[Title/Abstract]) AND (Cells[Title/Abstract] OR cell[Title/Abstract])). The search strategy was modified for each database individually.

### Selection process

Two authors independently screened the titles and abstracts of the records retrieved from the literature search according to our prespecified inclusion criteria. Full texts of the eligible records were obtained for further screening by another two authors. Any discrepancy between the authors in these two screening steps was resolved by a third reviewer.

### Data extraction

Data were extracted to structured excel sheets by two independent authors and disagreements between them were resolved through discussion with a third author.

The data were (1) characteristics of the included studies, including setting, design, intervention details, and summary of main findings; (2) demographic and baseline characteristics of the studies population; (3) quality assessment information (4) safety and efficacy endpoints.

### Risk of *bias* assessment

Two authors evaluated the quality of the included RCTs independently using the domains of Cochrane Collaboration’s tool for assessing risk of bias 1. We assessed the presence of bias in selection, performance, detection, attrition, and reporting, then classified the risk of these biases as high, low, or unclear^23^. The overall quality of each study was categorized into good, fair, and poor quality. Disagreements between the two authors were discussed and resolved with a third author.

### Effect measures

We included the following outcomes in our meta-analysis:

1. Clinical outcomes

**The primary outcome**:

- Expanded Disability Status Scale (EDSS) changes from baseline to 2, 6, and 12 months. An increased score indicates worsening of patient disability^25^.

**The secondary outcomes**:

 − Timed 25 Foot Walk (T25-FW) change at 6 months to assess the effect on motor performance. Better walking ability is associated with lower scores^26^
 − Change in the Nine-hole peg test (9-HPT) at 6 months that measures finger dexterity with lower scores indicating improvement^27^
 − Paced auditory serial addition test (PASAT-3) at the last follow-up to assess the effect on cognitive functions indicating improvement with increased scores^28^
 − Number of relapses evaluated in 6 months of follow-up.

2. Radiological outcomes

 − Change in the volume of MRI T2 weighted lesions at the end of treatment.
 − Change in the number of MRI T2-weighted lesions at 12 months.
 − Change in the number of gadolinium-enhancing lesions (GELs) at the end of the treatment period.

3. Safety endpoints

- We analyzed the reported incidence of headache, anemia, gastrointestinal disturbance, blood and lymphatic system disorders, total infections, administration site adverse events (AEs), and serious AEs.

Clinical and radiological outcomes were continuous data that were pooled as mean change from baseline (MC) and standard deviation (SD) of mean change. On the other hand, safety endpoints were dichotomous variables and were extracted as frequency of events in the total patients’ number in the group.

### Calculating the missing data

When the mean change from baseline to the time point of measurement of the outcome wasn’t reported, it was calculated by subtracting the pre-intervention values from the post-intervention values of the outcomes^29^.

When the standard deviations (SD) of mean change weren’t provided in the included studies, we calculated these SD values using Cochrane Handbook for Systematic Reviews of Interventions methods^29^. We also followed Wan et al.^30^ method to calculate mean and SD from median and (range or interquartile range), if provided.

### Statistical analysis

Outcomes reported by two or more studies were included in our analysis. We conducted the meta-analysis using Cochrane systematic review software Review Manager Version 5.4 for windows for continuous data; and OpenMeta Analyst software for windows for dichotomous data. We presented Continuous variables as mean differences (MDs) with 95% confidence intervals (CIs) and dichotomous variables as relative risks (RRs) with 95% CIs. We considered data statistically significant when *p* value is ≤0.05.

### Assessing the heterogeneity

The heterogeneity of the studies and subgroups was evaluated by visualizing the forest plot based on the Cochrane Q and I-square tests. We set a *P* value of *P* < 0.1 and I^2^ ≥ 50% as the significance level for assessing heterogeneity^29^. We pooled data under a random-effects model due to the variation in the patients’ characteristics and procedural aspects among the included studies.

### Subgroup analysis

We conducted the subgroup analyses for EDSS change from baseline based on (A) the time of assessment at 2, 6, and 12 months after the intervention; (B)baseline EDSS ≤ 6.5 or > 6.5 (EDSS of 6.5 correlates with walking ability of 20 m with two aids^25^); (C) the administrated dose of stem cells in each study, either low doses (≤ 2 × 10^6^ cells/kg) or higher doses (≥ 3 × 10^6^ cells/kg); (D) the sources of the transplanted stem cells (adult or embryonic origin) and (E) whether the control was a placebo or active treatment.

Subgroups from B–D included EDSS change from baseline to the end of patients follow-up (last assessment).

### Sensitivity analysis

We ran a sensitivity analysis using the leave-one-out procedure which includes conducting multiple meta-analyses for the outcomes and excluding a single study in each scenario to investigate the impact of these studies on the overall effect size. By this method, we ensure the statistical robustness of our results and that the results of our meta-analysis were not affected by any of the individual studies. OpenMeta Analyst software was used to perform the sensitivity analysis.

### Publication bias assessment

We planned to explore the publication bias in the included studies using Egger’s method depending on funnel plot asymmetry^24^.

## Results

### Study selection

From the initial literature search, we retrieved relevant 3948 records from PubMed, Web of Science, Scopus, and the Cochrane Library. After the title and abstract screening of them we screened the full text of 295 articles. Only nine studies met our criteria^31–39^. Figure 1 shows the PRISMA flow diagram of our search and selection process.

### Study characteristics

The nine studies were RCTs and enrolled a total of 422 multiple sclerosis patients. All studies were parallel in design except 4 studies were cross-over RCTs^31,32,34,39^. These cross-over trials were reviewed up to the point of cross-over. All studies infused stem cells intravenously except Petrou et al. that included an additional intrathecal SCT subgroup^32^. This study showed that intrathecal SCT was more effective than intravenous SCT, but we pooled the data of both routes as single study data. Of the included studies, only two studies used autologous hematopoietic SCT (AHSCT) in addition to immune ablative regimen prior to the transplantation^37,38^. Burt et al. compared SCT to DMTs (natalizumab, fingolimod, and dimethyl fumarate) in RRMS patients^37^, and Mancardi et al., compared SCT to mitoxantrone in relapsing and progressive MS patients^38^.

Supplementary Table S1 summarizes the characteristics of the included trials, Table 1 shows the demographic and baseline characteristics of these studies’ population, and Supplementary Table S3 shows efficacy endpoints reported at 6 months.

**Table 1:** Baseline and demographic data of the included population.

Study ID	Total Sample Size,n (I/C)	Age, y, mean (SD)		Males, % (I/C)	Disease subtypes, %		Disease Duration, y, mean(SD)		Baseline EDSS, mean(SD)		Baseline GELs, mean(SD)	
		I	C		I	C	I	C	I	C	I	C
Li et al.^35^	23 (13/10)	14.7 (5.6)	39.4 (3.8)	30/30.8	RRMS (69.2) SPMS (30.8)	RRMS (70) SPMS (30)	2.9(0.9)	2.57(0.8)	6.98(1.2)	6.02(1.6)	NA	NA
Llufriu et al.^34^	9 (5/4)	35.6 (8.71)	38.25 (9.11)	40/0	RRMS (100)	RRMS (100)	8.68(2.79)	7.98(2.46)	4.2(1.3)	3.88(0.85)	4.6(9.74)	4.75(7.63)
Lublin et al.^33^	16 (12/4)	48.88 (5.61)	46.75 (3.49)	25/50	RRMS(58.33) SPMS (41.67)	RRMS (75) SPMS (25)	10.84(6.52)	12.8(7.12)	4.94(1.1)	4(0)	0.125(0.25)	0(0)
Mancardi et al.^38^	21 (9/12)	36	35	44.44/25	RRMS(22) SPMS (78)	RRMS (42) SPMS (50) RPMS (8)	NA	NA	6.25(0.32)	6(0.29)	NA	NA
Ferna´ndez et al.^36^	30 (19/11)	46.21 (8.73)	46.3 (8.9)	31.58/27.27	SPMS (100)	SPMS(100)	16.96(7.42)	18.9(7.3)	7.63(0.6)	7.64(0.98)	1.32(2.32)	0.82(1.17)
Burt et al.^37^	110 (55/55)	35.6 (8.4)	35.6 (8.2)	38/34	RRMS (100)	RRMS(100)	5.3	7.1	3.4(1.2)	3.3(1)	4.5 (8.2)	4.9 (8.4)
Petrou et al.^32^	48 (32/16)	48.24 (8.84)	45.89 (10.9)	50/75	SPMS (87.5) PPMS (12.5)	SPMS (81.25) PPMS (18.75)	11.59(6.96)	14.94(8.27)	6(0.61)	5.66(1.08)	0.69(1.46)	0.69(1.96)
Uccelli et al.^31^	144 (69/75)	39·7 (6·35)	38·3 (6·97)	41/39	RRMS (67) SPMS (23) PPMS (10)	RRMS (64) SPMS (23) PPMS (13)	NA	NA	4.5(1.89)	4.67(1.89)	0.67(1.5)	0.67(1.5)
Nabavi et al.^39^	21 (12/9)	32.25 (3.82)	39.33 (11.21)	25/22.22	RRMS (88.3) SPMS (16.67)	RRMS (44.4) SPMS (33.3) PPMS (22.2)	8.92(3)	10.78(3.27)	5(1.28)	5.56(1.29)	NA	NA

*I* intervention arm (stem cell transplantation), *C* control, *SD* standard deviation, *RRMS* relapsing–remitting multiple sclerosis, *SPMS* secondary-progressive multiple sclerosis, *PPMS* primary-progressive multiple sclerosis, *RPMS* relapsing-progressive multiple sclerosis, *NA* not available.

### Risk of bias within studies

**Figure 2:**
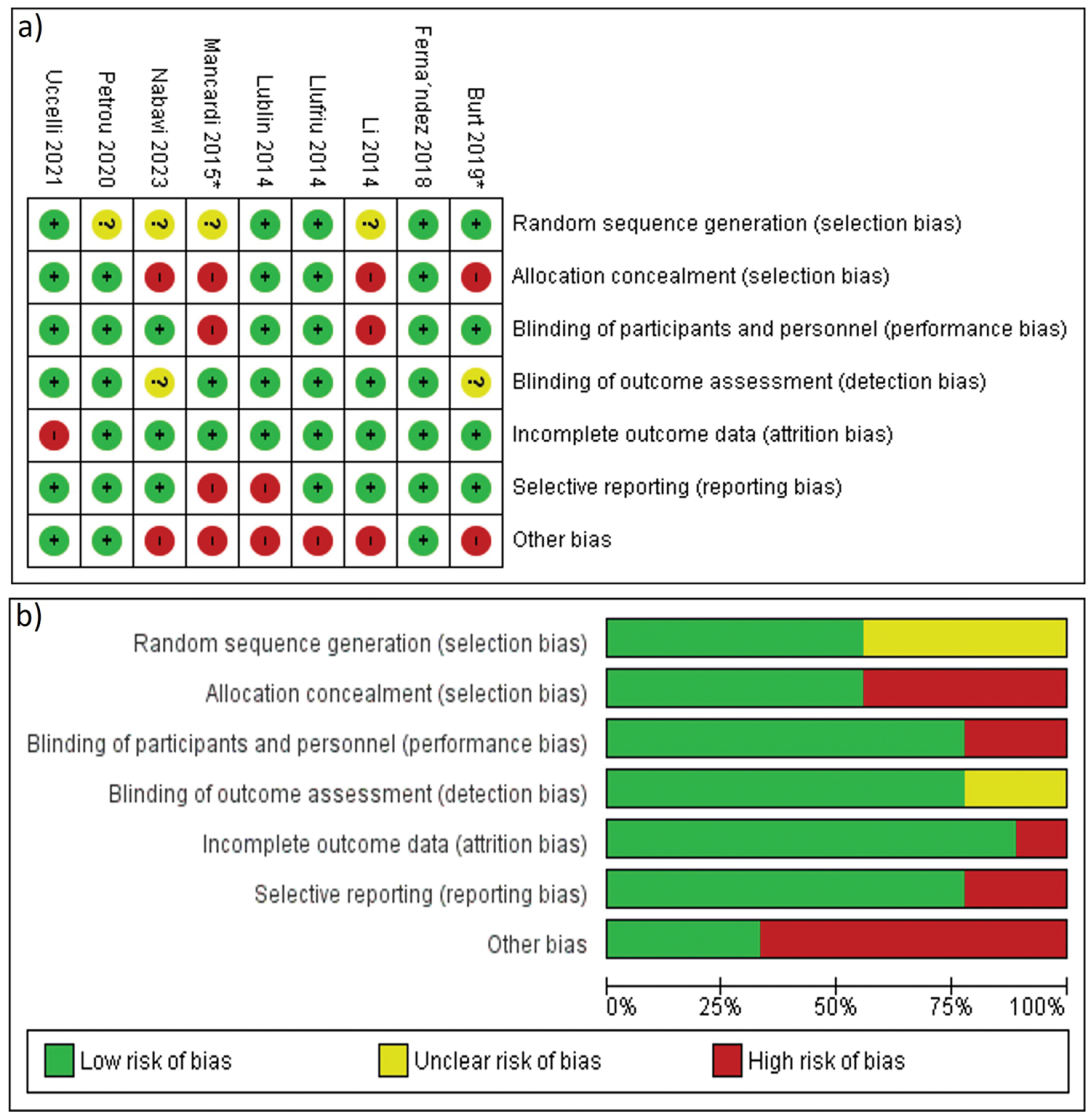
Risk of bias assessment: (**a**) Risk of bias summary, (**b**) Risk of bias graph.

We assessed seven domains in each study according to The Cochrane Collaboration’s tool for assessing risk of bias 1. The 9 studies were randomized but 4 studies^32,35,38,39^ didn’t clarify the methods of random sequence generation. 6 RCTs confirmed concealment of patients’ allocation to the intervention^31–34,36,37^. Blinding of the outcome assessors was clearly stated in all studies except Nabavi et al.^39^ but blinding of participants and personnel wasn’t fulfilled in three studies^35,37,38^. The reasons for incomplete outcome data are related to the treatment in Uccelli et al.^31^ and the reasons weren’t clearly described in Burt et al^37^. Two studies reported the outcomes in an incomplete way that limited their inclusion in the meta-analysis inducing a reporting bias^33,38^. The overall quality of the studies was good for 2 studies^32,36^, fair for 3 studies^31,34,37^, and poor for 4 studies^33,35,38,39^. Figure 2 shows the risk of bias summary and graph.

### Meta-analysis results

After analyzing the efficacy and safety outcomes for all studies collectively, we subdivided the results into studies that used immunosuppression before AHSCT^37,38^ and studies that transplanted mesenchymal stem cells (MSCs) without immunosuppression^31–36,39^ to minimize the procedural variations among the included trials.

#### Clinical outcomes

##### Effect on patients disability

The majority of the studies^31–37,39^ reported EDSS change for 211 patients in stem cell transplantation (SCT) arm and 176 controls. Because the time of reporting this outcome varied among the studies, we analyzed EDSS change at the last follow-up reported by each study. Our analysis showed nonsignificant difference between SCT group and the control group (MD = − 0.48, 95% CI [− 1.11, 0.14], *p* = 0.13). There was great heterogeneity between studies (χ2 = 116.74, df = 7, *p* < 0.00001, I^2^ = 94%), so we pooled the data under the random-effects model (Table 2 and Supplementary Figure S1).

**Table 2:** Subgroup analyses of EDSS.

Outcome	Studies No	Patients No. (I/C)	Analysis Model	Effect of estimate, MD, 95% CI	*P*-value	Heterogeneity, I^2^ (*p* value)
EDSS at last follow-up	8	211/176	RE	− 0.48 [− 1.11, 0.14]	0.13	94% (*p* < 0.00001)
EDSS (2 months)	2	25/14	RE	− 0.57[− 1.08, − 0.06]	0.03	38% (*P* = 0.2)
EDSS (6 months)	7	194/171	RE	− 0.48 [− 0.98, 0.03]	0.07	91% (*p* < 0.00001)
EDSS (12 m)	3	82/69	RE	− 1.04 [− 2.12, 0.04]	0.06	95% (*p* < 0.00001)
Baseline EDSS ≤ 6.5	6	179/155	RE	− 0.41 [− 1.11, 0.29]	0.25	94% (*p* < 0.00001)
Baseline EDSS > 6.5	2	32/21	RE	− 0.68 [− 2.68, 1.32]	0.5	97% (*p* < 0.00001)
EDSS for low doses-studies	6	177/162	RE	− 0.31 [− 1.00, 0.38]	0.37	95% (*p* < 0.00001)
EDSS for high doses-studies	3	34/25	RE	− 0.57 [− 1.94, 0.80]	0.41	89% (*p* = 0.00001)
EDSS for embryonic stem cells	2	25/14	RE	− 1.01 [− 2.46, 0.45]	0.17	88% (*p* = 0.004)
EDSS for adult stem cells	6	186/162	RE	− 0.32 [− 1.02, 0.38]	0.37	94% (*p* < 0.00001)
EDSS: the control is placebo	5	143/114	RE	− 0.09 [− 0.46, 0.28]	0.62	63% (*p* = 0.03)
EDSS: the control is active drug	3	68/62	RE	− 1.21 [− 1.98, − 0.43]	0.002	88% (*p* = 0.0002)

*I* intervention, *C* control, *MD* mean difference, *CI* confidence interval, EDSS expanded disability status scale, *RE* random effects.

The subgroup analysis of the studies that used MSCs without immunosuppression also showed nonsignificant improvement (MD = − 0.3, 95% CI [− 0.87, 0.27], *p* = 0.3). However, Burt et al^37^. that used immunosuppression before AHSCT revealed significant EDSS reduction (Supplementary Figure S1).

The results remained nonsignificant after the leave-one-out sensitivity analysis (Supplementary Figure L1).

### Subgroup analyses for EDSS

#### EDSS at different timepoints

##### EDSS at 2 months

**Figure 3:**
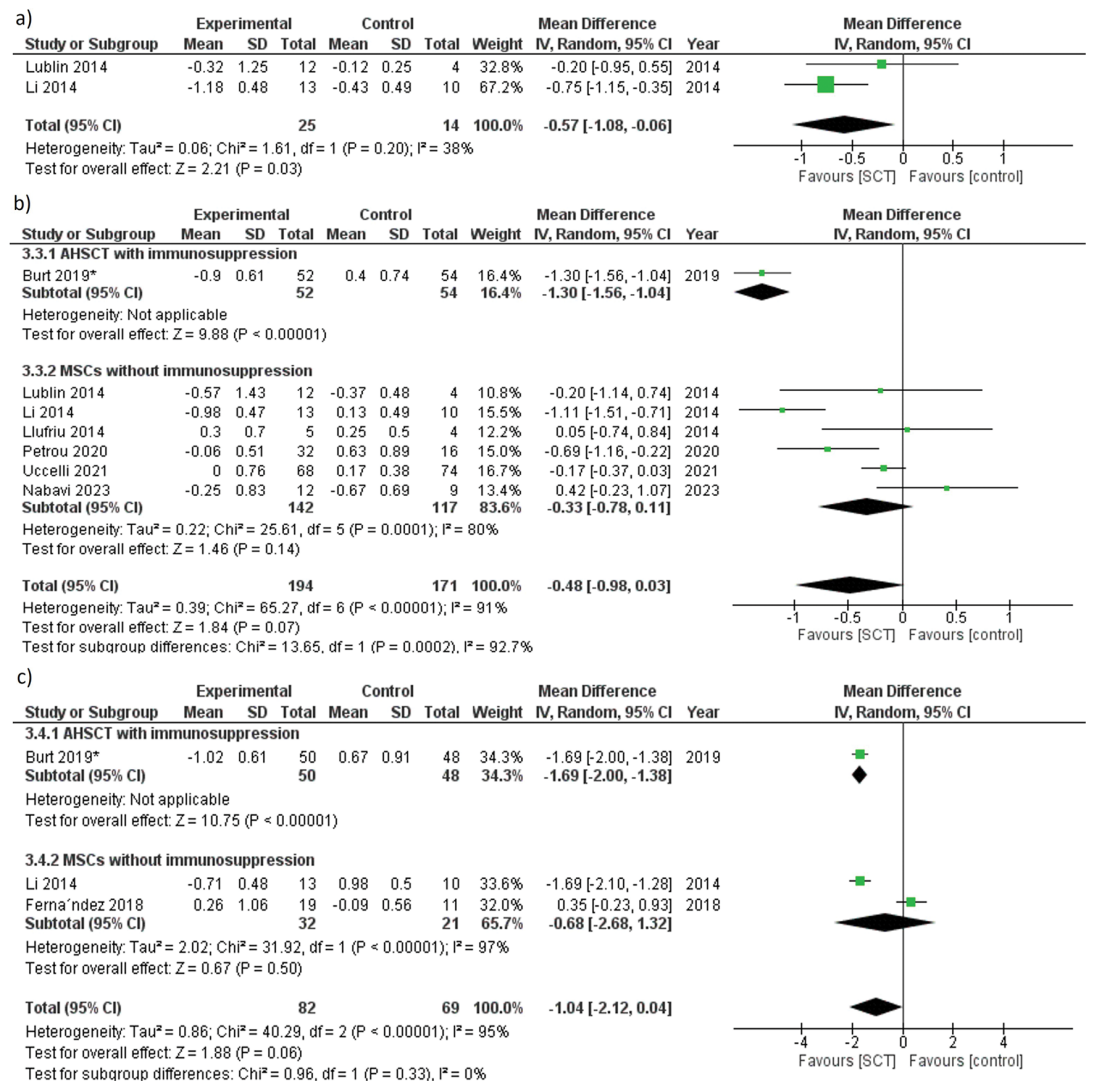
Forest plot of EDSS change from baseline at (**a**) 2 months, (**b**) 6 months, (**c**) 12 months.

The heterogeneity within the studies was not significant (χ2 = 1.61, df = 1, *p* = 0.2, I^2^ = 38%), and we adopted a random effect model. The reduction of EDSS in SCT group was significantly greater than the control group (MD =  − 0.57, 95% CI [− 1.08, − 0.06], *p* = 0.03) (Table 2 and Fig. 3a).

##### EDSS at 6 months

Adopting the random-effects model, the heterogeneity between the studies was significant (χ2 = 65.27, df = 6, *p* < 0.00001, I^2^ = 91%), and SCT showed nonsignificant improvement of EDSS compared to the control (MD =  − 0.48, 95% CI [− 0.98, 0.03], *p* = 0.07) (Table 2 and Fig. 3b). MSCs without immunosuppression also resulted in nonsignificant EDSS reduction at 6 months (MD = − 0.33, 95% CI [− 0.78, 0.11], *p* = 0.14) (Fig. 3b).

The effect estimate changed to (MD =  − 0.62,95% CI [− 1.14, − 0.09], *p* = 0.02) favoring SCT over the control after excluding Nabavi et al.^39^ from the analysis (Supplementary Figure L2 and Table L1).

##### EDSS at 12 months

We adopted the random-effects model because heterogeneity was significant, and the difference between the SCT group and the control was not significant at 12 months for both collective studies analysis and studies used MSCs without immunosuppression (*p* = 0.06 and *p* = 0.5, respectively). However, the study that used AHSCT plus immunosuppression^37^ showed significant improvement in patients disability (*p* < 0.00001) (Table 2 and Fig. 3c).

After performing a sensitivity analysis by excluding Ferna´ndez et al.^36^, the results changed from nonsignificant to significant improvement in SCT arm (MD = − 1.69, 95% CI [− 1.94, − 1.44], *p* < 0.00001) (Supplementary Figure L3 and Table L1).

#### EDSS improvement according to baseline EDSS

We compared the effect of SCT on patients’ disability depending on baseline EDSS. Six studies^31–34,37,39^ included 334 MS patients with baseline EDSS ≤ 6.5, while two studies^35,36^ included 53 patients with baseline EDSS > 6.5. Using a random effects model, both subgroups showed significant heterogeneity (*p* < 0.00001 and *p* < 0.00001). Both subgroups revealed nonsignificant effect of SCT on EDSS, (MD = − 0.41, 95% CI [− 1.11, 0.29], *p* = 0.25) for baseline EDSS ≤ 6.5 subgroup and (MD = − 0.68, 95%CI [− 2.68, 1.32], *p* = 0.5) for baseline EDSS > 6.5 subgroup (Table 2 and Supplementary Figure S2).

#### EDSS according to the doses of stem cells

We pooled data of EDSS change from baseline to the last assessment time under a random-effects model, and the differences were nonsignificant for both low and high doses subgroups, (MD = − 0.31, 95% CI [− 1, 0.38], *p* = 0.37) and (MD = − 0.57, 95% CI [− 1.94, 0.8], *p* = 0.41), respectively. The studies of both subgroups showed significant heterogeneity (I^2^ = 95%, *p* < 0.00001) for the low doses subgroup, and (I^2^ = 89%, *p* = 0.0001) for the high doses subgroup (Table 2 and Supplementary Figure S3).

#### EDSS analysis with stem cells of adult and embryonic origin

Adopting a random-effects model, stem cells from embryonic as well as adult origin showed nonsignificant effect on EDSS (*p* = 0.17, and *p* = 0.37, respectively), With significant heterogeneity among the studies (I^2^ = 88%, *p* = 0.004), and (I^2^ = 94%, *p* < 0.00001), respectively (Table 2 and Supplementary Figure S4).

#### EDSS analysis according to the control group

We pooled data using a random-effects model. Five studies^31–33,36,39^, in which placebo was the control, showed substantial heterogeneity (I^2^ = 63%, *p* = 0.03) and the difference between SCT and placebo was not significant (MD = − 0.09, 95% CI [− 0.46, 0.28], *p* = 0.62). Three studies^34,35,37^, in which the control was active treatment, showed significant reduction of EDSS with SCT compared to the active drugs (MD = − 1.21, 95% CI [− 1.98, − 0.43], *p* = 0.002) and the heterogeneity was significant (I^2^ = 88%, *p* = 0.0002) (Table 2 and Supplementary Figure S5).

### Number of relapses during 6 months of follow-up

Only two studies^32,34^ reported the number of relapses in the 6 months following the intervention. Under a random-effects model, the heterogeneity was moderate (*p* = 0.14, I^2^ = 53%), and the decrease in relapses number was nonsignificant (*p* = 0.23) (Supplementary Figure S6).

### Timed-25 foot walk (T25-FW) change at 6 months

**Figure 4:**
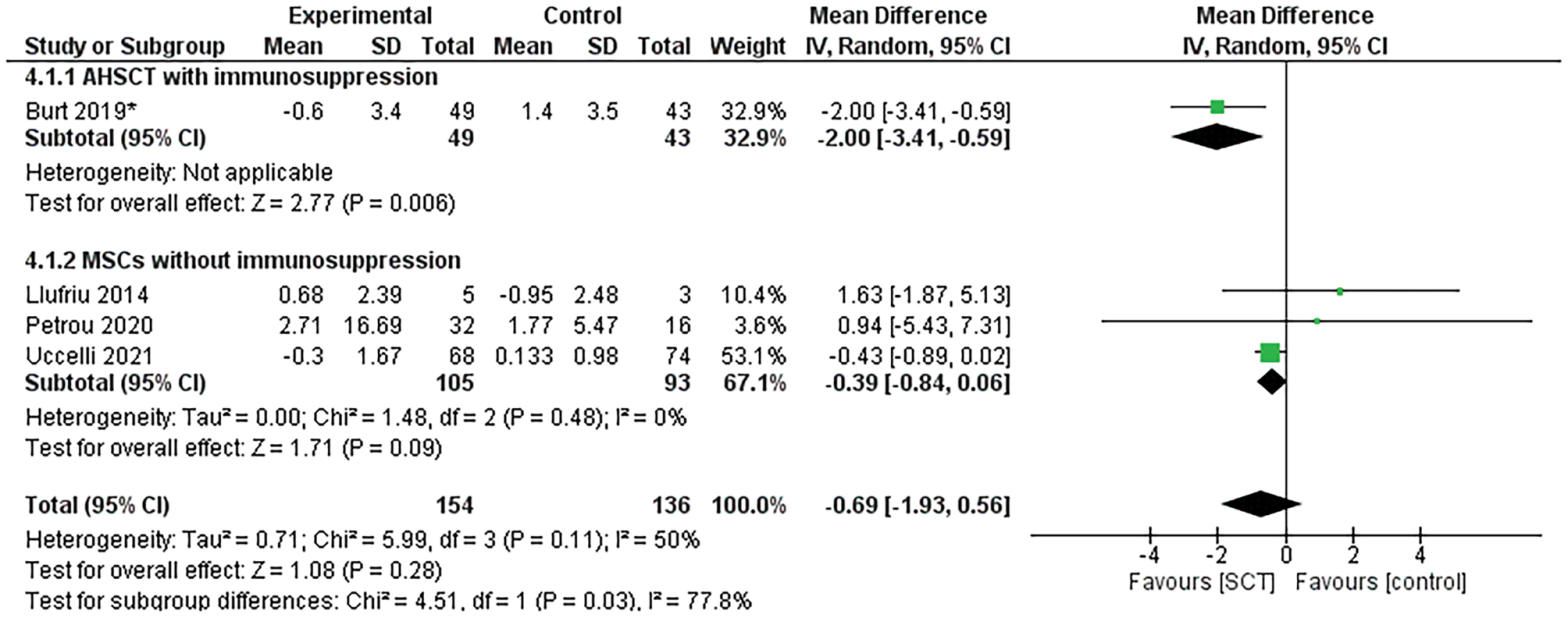
Forest plot of T25-FW change from baseline.

Four studies^31,32,34,37^ assessed T25-FW in 154 and 136 patients in the SCT and control groups, respectively. We pooled data under a random-effect model, and heterogeneity was moderate (χ2 = 5.99, df = 3, *p* = 0.11, I^2^ = 50%). SCT resulted in a nonsignificant improvement in patients’ T25-FW scores compared to the control group (MD = − 0.69, 95% CI [− 1.93, 0.56], *p* = 0.28), as shown in Fig. 4.

In the studies that included mesenchymal SCT without immunosuppression, the improvement in patients’ T25-FW scores after SCT was not significant (MD = − 0.39, 95% CI [− 0.84, 0.06], *p* = 0.09), but T25-FW significantly improved in the study that used AHSCT and immunosuppression^37^ (*p* = 0.006). Figure 4 demonstrates these analyses. The *p* value of the results didn’t change after the one-study-removed sensitivity analysis (Supplementary Figure L4).

### Change in Nine-hole peg test (9-HPT) at 6 months

9-HPT was evaluated in four RCTs^31,32,34,37^. We used a random-effects model because heterogeneity was significant (*p* = 0.0003, I^2^ = 84%). 9-HPT showed nonsignificant improvement in the collective analysis and the sub-analysis of MSCs without immunosuppression. However, Burt et al.^37^. revealed a significant improvement (*p* < 0.00001) (Supplementary Figure S7). The results remained nonsignificant after sensitivity analysis (Supplementary Figure L5).

### Change of Paced auditory serial addition test (PASAT-3) score

We pooled PASAT-3 scores assessed at the end of treatment in four trials under a random-effects model^31,34,36,37^. Heterogeneity was minimal (*p* = 0.35, I^2^ = 9%), and the differences were nonsignificant in the collective analysis and the sub-analysis of autologous and mesenchymal SCT (*p* = 0.35, *p* = 0.96, and *p* = 0.31, respectively) (Supplementary Figure S8). Effect estimate remained nonsignificant after one-study-removed sensitivity analysis (Supplementary Figure L6).

#### Radiological outcomes

##### Change in the volume of MRI T2-weighted lesions

**Figure 5:**
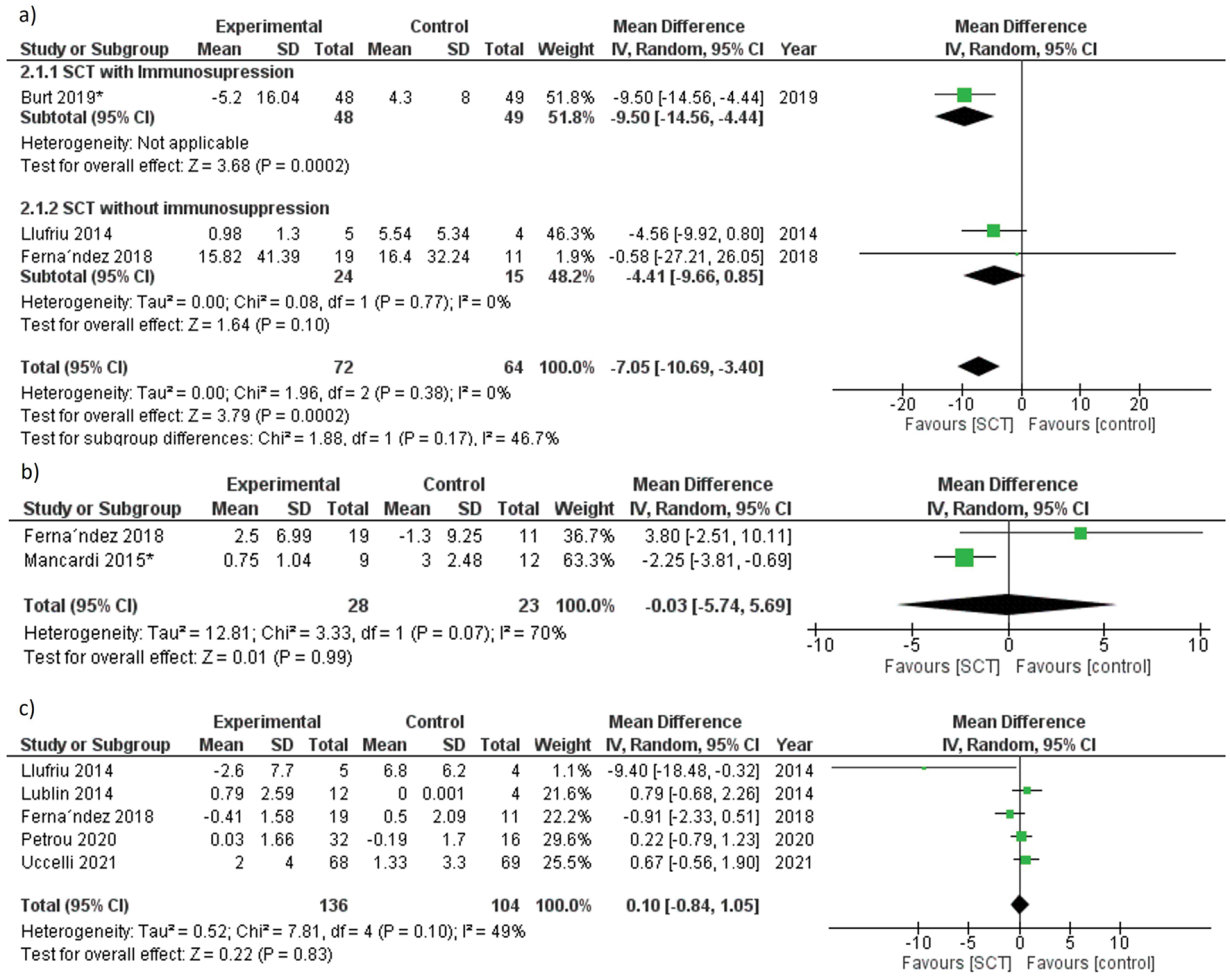
Forest plot of radiological outcomes change from baseline (**a**) MRI T2-weighted lesions volume at the end of treatment, (**b**) MRI T2-weighted lesions number at 12 months, (**c**) number of GELs at the end of treatment. *the study used immunosuppression before AHSCT.

We analyzed the change in brain lesion volume from baseline to the end of the follow-up. Data were pooled under a random-effects model, heterogeneity was absent (*p* = 0.38, I^2^ = 0%). Our analysis revealed a significant reduction in T2 lesions volume (MD = − 7.05, 95% CI [− 10.69, − 3.4], *p* = 0.0002). In the studies that used MSCs without immunosuppression, the reduction of brain lesions volume was nonsignificant (*p* = 0.1) (Fig. 5a).

The results became nonsignificant and changed to (MD = − 4.41, 95% CI [− 9.66, 0.85], *p* = 0.1) after a sensitivity analysis performed by excluding Burt et al.^37^ (Supplementary Figure L7 and Table L1).

##### Change in the number of MRI T2-weighted lesions

Adopting a random-effects model, the studies showed substantial heterogeneity (*p* = 0.07, I^2^ = 70%). And the differences between SCT and the control after 12 months were nonsignificant (*p* = 0.99) (Fig. 5b).

##### Change in the number of gadolinium-enhancing lesions (GELs) at the end of the treatment

Five studies^31–34,36^ assessed this outcome. Four studies reported the change of GELs number from baseline at 6 months except Ferna´ndez et al.^36^ at 12 months. We pooled data under a random-effects model and heterogeneity was not significant (χ2 = 7.81, df = 4, *p* = 0.1, I^2^ = 49%). Our analysis revealed nonsignificant differences in GELs number change (*p* = 0.83) (Fig. 5c). The results didn’t change after sensitivity analysis (Supplementary Figure L8).

### Incidence of adverse events (AEs)

Seven studies^31–34,36–38^ reported adverse events that occurred during the follow-up period. Two studies^35,39^ didn’t provide data about AEs. Nabavi et al. mentioned only pain at the site of bone marrow aspiration^39^. Our analysis revealed that the difference was nonsignificant between SCT and the control group regarding the incidence of most AEs. Administration-related AEs, including infusion site swelling, hematoma, and pain, were significantly more common in the SCT group compared to the control (N = 25, RR = 2.55, 95% CI [1.08, 6.03], *p* = 0.034). On the other hand, the SCT group had a lower incidence of total infections (any infection during the follow-up period, including viral infections, respiratory, urinary infections, scabies, and other infestations) than the control group (N = 60, RR = 0.58, 95% CI [0.37, 0.9], *p* = 0.02). Regarding the use of immunosuppression, AHSCT combined with immunosuppression was significantly associated with a higher incidence of blood and lymphatic system disorders (N = 16, RR = 2.33, 95% CI [1.23, 4.39], *p* = 0.009). The analyses of the adverse events are shown in Table 3 and Supplementary Figures S9–S14. No transplant-related mortality was noted in all trials during the follow-up period, except for two unrelated deaths compacted by Ferna´ndez et al. in the placebo arm (one due to choking while feeding and the other due to respiratory infection)^36^.

**Table 3:** The analysis of the adverse events in all studies.

Adverse events (AEs)	Studies No	I/C For SCT arm	I/C For control	Model	Effect of estimate, RR, 95% CI	*P* value	Heterogeneity, I^2^% (*p* value)
Serious AEs	6	9/150	12/122	RE	0.64 [0.16, 2.58]	0.53	41.95% (*p* = 0.13)
Administration-related AES	2	19/81	6/79	RE	2.55 [1.08, 6.03]	0.034	0% (*P* = 0.6)
Headache	2	14/44	8/20	RE	0.79 [0.41, 1.5]	0.47	0% (*P* = 0.53)
Total infections	3	22/110	38/103	RE	0.58 [0.37, 0.9]	0.02	0% (*P* = 0.44)
Gastrointestinal disorders	3	18/83	10/91	RE	1.72 [0.37, 7.87]	0.49	69.63% (*p* = 0.04)
Blood and lymphatic system disorders	3	12/130	8/138	RE	1.75 [0.59, 5.14]	0.31	25.83% (*P* = 0.26)

*CI* confidence interval, *RE* random effects.

### Publication bias

We examined the publication bias among the studies that reported the effect of SCT on patients’ disability using the funnel plot test. Although there was funnel plot asymmetry, the test isn’t reliable because the included studies were less than ten studies^24^ (Supplementary Figure S15).

## Discussion

Most of multiple sclerosis (MS) patients suffer from sensory, motor, and cognitive impairment. However, no curative treatment for this disease is available up till now. The approved disease modifying therapies (DMTs) for relapsing remittent MS showed effective short-term results, but the benefit for progressive subtypes is limited with fewer treatment options^40^. On the other hand, autologous hematopoietic stem cell transplantation (AHSCT) could achieve complete suppression of MS disease activity in 70–80% of patients for 4–5 years, which is superior to other MS therapeutic options^14^. AHSCT also showed promising results with aggressive MS and patients refractory to DMTs^41^. Also, AHSCT is more frequently used to treat aggressive MS than allogeneic hematopoietic SCT, because allogenic hematopoietic SCT is associated with a risk of graft-versus-host disease that increases patients morbidity and mortality rates^42^. On the other hand, various preclinical studies using animal models revealed that mesenchymal stem cells (MSCs) could ameliorate MS symptoms and delay disease progression^43^. In recent human clinical trials, MSCs improved MS symptoms and showed immunoregulatory and anti-inflammatory functions without the need for intense immunosuppression^18^.

To assess the effect of SCT on MS, we included nine RCTs in our meta-analysis with an overall population of 422 patients. Our results revealed that SCT was significantly superior to the control in improving patients EDSS at 2 months and reducing MRI-T2 weighted lesions volume. However, improvement in EDSS in other clinical outcomes was not significant. Regarding SCT safety, adverse events showed nonsignificant differences except for site reactions. However, patients who received SCT significantly experienced a lower incidence of total infections.

The primary outcome of our meta-analysis is EDSS, the most commonly used standardized and validated tool for disability progression^44^. Initially, we pooled EDSS changes from baseline to the last follow-up reported by the studies. Because it was reported that follow-up period variation represents a potential confounder of disability outcomes, we conducted a subgroup analysis based on the follow-up period^45^. Our results of these subgroups significantly confirmed the effect of SCT on delaying patients’ disability progression at 2 months and at 6 months (after the sensitivity analysis), agreeing with a previous meta-analysis on AHSCT^46^. Although EDSS change was not significant at 6 and 12 months, the sensitivity analysis showed a significant change in the effect size. EDSS at 6 months significantly favored SCT after excluding Nabavi et al., that revealed a non-remarkable difference between both arms^39^. Additionally, EDSS at 12 months significantly changed by excluding Fernández et al.^36^, that included only SPMS patients with longer disease duration and deteriorating patients disability affecting the analysis results. Previous literature found that SCT is more effective with RRMS than progressive MS and linked this to the lower baseline EDSS and early disease stage^10,46,47^. However, we performed a subgroup analysis based on baseline EDSS scores and detected nonsignificant effects on disability progression. The results of the other subgroup analyses regarding the source and the dose of stem cells supported the findings of a previous meta-analysis of preclinical trials of nonsignificant effects of these procedural aspects on disease progression^43^.

We assessed the effect of SCT on patients walking ability by the T25-FW test that is a reliable tool for the short and long-terms^26^. The analysis of T25-FW showed nonsignificant improvement in the collective analysis and in patients who received MSCs without immunosuppression. However, patients received AHSCT preceded by immunosuppression showed significant improvement. The results of T25-FW scores analysis is consistent with the EDSS analysis because both endpoints are affected by patients’ lower extremity disability. Regarding the radiological outcomes, the remission in MRI lesion volumes reflects the suppression of the inflammatory process in the brain and prevention of further disease progression, as explained by Genovese et al.^48^ Also, the reduction of lesion volume is consistent with EDSS improvement according to a study that proved a positive correlation between MRI lesion volume and patients’ disability^49^. But after performing a sensitivity analysis by excluding Burt et al., the reduction in MRI-T2 lesion volume became nonsignificant indicating that Burt et al. may account for the significant improvement ^37^.

The overall improvement in patients upper extremity function was nonsignificant, and none of the trials assessed this outcome showed a significant change in 9-HPT; however, Burt et al.^37^ revealed a significant improvement in 9-HPT that can be justified by including only RRMS patients with low baseline EDSS compared to other trials. The presence of ceiling and floor effects in 9-HPT is a factor that may have affected this outcome. As reported, 9-HPT isn’t sensitive enough for detecting hand dexterity improvement in low or high disability patients^50^.

In terms of SCT safety, nonsignificant adverse events observed in the studies included headache and gastrointestinal disturbances. MS patients are known to suffer from a high incidence of infections, especially respiratory infections that may be complicated by death^51^. Surprisingly, in this meta-analysis SCT patients significantly experienced a lower incidence of total infections compared to their control, showing a protective effect against those infections. This can be explained by releasing antimicrobial substances from stem cells (particularly MSCs) such as beta-defensins, cathelicidin LL-37, and other peptides. Other functions of stem cells including, immunomodulatory, anti-inflammatory, and regenerative effects also contribute to fighting pathogens and combacting infections-related tissue damage^52^. Regarding transplant-related mortality, our results of no deaths in the follow-up period are similar to that reported by a previous meta-analysis that included 133 patients^53^. Treatment-related mortality of AHSCT in MS has dropped from 3.6% (in studies before 2005) to 0.3% (in studies since 2005), and this reduction in mortality was more evident in the younger population^14^. Additionally, allogeneic stem cells transplantation was associated with improved mortality rates in recent years in treating different autoimmune diseases^54^. Contrary to this evidence, a recent meta-analysis of AHSCT detected a higher transplant-related mortality of 4% in a 5 year follow-up duration^55^. However, other studies estimated the mortality rates during shorter post-transplant period, which indicates a need for standardizing the duration of assessing such an important endpoint. Another meta-analysis of AHSCT^56^ revealed a significant association between patients’ mortality and both MS clinical subtypes and baseline EDSS. Lower mortality rates were observed in RRMS patients, and higher baseline EDSS were linked to higher mortality rates^56^.

Regarding the studies that included an immune ablative regimen before AHSCT, the reported results showed a significant improvement in EDSS, T25-FW, 9HPT, and lesions volume in Burt et al.^37^ and a significant reduction in lesions number in Mancardi et al.^38^. Immunoablation followed by SCT has been considered in several autoimmune diseases to induce sustained remission. High-dose chemotherapy eradicates the autoreactive immunologic memory, and SCT following it would regain the immunologic self-tolerant state causing long-term remission of autoimmune diseases^57^. Future trials are needed to assess the long-term effects of immunoablation before SCT.

Finally, our meta-analysis provides an up-to-date valid conclusion on SCT efficacy and safety based on RCTs. We depended on a well-defined search strategy and criteria to include all eligible studies. We prepared this study following the PRISMA checklist and performed all the steps according to the Cochrane Handbook for Systematic Reviews of Interventions. We included cross-over trials to increase the sample size of the analysis to get credible results, and these studies were included until the cross-over point to avoid the carry-over effect in such trials. We analyzed all possible efficacy and safety outcomes to provide a comprehensive view of the role of SCT in MS.

### Limitations

Our meta-analysis faced some limitations. First of all, we couldn’t provide a quantitative comparison between SCT and the approved DMTs for MS because few studies included DMTs as the control. And the number of studies was inadequate for conducting a comprehensive quantitative analysis of the efficacy of immunosuppression before SCT. Our study was also limited by the short follow-up periods (maximally 12 months) in the included trials, so the long-term effect of SCT is still questionable. Moreover, pooling the results of the cross-over studies up to the point of cross-over resulted in a further limitation in detecting long-term results.

Furthermore, heterogeneity was evident among the studies in most outcomes. This heterogeneity is attributed to the non-uniform patients’ characteristics and procedural parameters. Patients in the trials had variable disease duration, disease course, baseline clinical measures, and previous use of DMTs. Regarding the disease course, most studies included progressive and relapsing MS patients without reporting the outcomes of each subtype separately. This hindered us from performing a subgroup analysis depending on this variable, particularly in determining the effect of SCT on progressive MS that needs to be explored. Also, the studies showed variations in the transplantation procedure that could have contributed to this heterogeneity, including immunity suppression before the transplantation, the doses and sources of stem cells, and the routes of stem cell infusion. Particularly, the lack of RCTs investigating AHSCT combined with immunosuppression in MS limited comparing it to MSCs and generalizing our results. The previous use of DMTs varied among the studies’ population, also the washout periods of these DMTs before patients inclusion weren’t stated in some trials and were inadequate in other trials which may have impacted the results. Finally, assessing publication bias wasn’t reliable because the pooled studies were less than ten.

### Recommendations

We recommend conducting future RCTs comparing SCT with the approved DMTs for more accurate and direct evidence. Also, comparing the transplantation of different sources of stem cells with and without immunosuppression is needed. Longer follow-up of RCTs will help to detect the long-term effect on disease progression and determine long-term safety concerns, particularly transplantation-related mortality. We also encourage RCTs to compare different routes of SCT, especially intrathecally, to determine the administration route that yields optimal results. Finally, detecting the effect of SCT on each MS clinical subtype separately is required to provide individualized treatment approaches.

## Conclusion

This meta-analysis showed that SCT improves multiple sclerosis patients’ disability at 2 months and reduces their brain lesions volume. SCT was tolerable and safe, with no mortality during the follow-up period. Patients who received MSCs significantly experienced local adverse events at the site of infusion. And in the studies that used AHSCT and immunosuppression, SCT patients significantly suffered from blood and lymphatic system disorders. However, we cannot generalize our results due to the sparse number of RCTs assessing AHSCT combined with immunosuppression for MS. We recommend conducting further RCTs of longer durations without cross-over, on specific subtypes of MS, using immunosuppression before the transplantation, and comparing SCT with approved DMTs to support evidence-based management. Including Naïve patients not previously treated with other DMTs will guarantee pure assessment of SCT safety and efficacy.

### Supplementary Information

Supplementary Information 1.

Supplementary Information 2.

## Data Availability

All data generated or analyzed during this study are included in this published article (and its Supplementary Information files).

## Abbreviations

MS: Multiple sclerosis
SCT: Stem cell transplantation
MSCs: Mesenchymal stem cells
DMTs: Disease modifying therapies
RCT: Randomized controlled trial
RRMS: Relapsing-remitting multiple sclerosis
SPMS: Secondary-progressive multiple sclerosis
PPMS: Primary-progressive multiple sclerosis
EDSS: Expanded disability status scale
T25-FW: Timed 25 Foot Walk
9-HPT: Nine-hole peg test
PASAT-3: Paced auditory serial addition test
MRI: Magnetic resonance imaging
GELs: Gadolinium-enhancing lesions
AEs: Adverse events
