# Supplementary material for: Efficacy and safety of stem cell transplantation for multiple sclerosis: a systematic review and meta-analysis of randomized controlled trials
Source: Sci Rep. 2024 May 31;14:12545. doi: 10.1038/s41598-024-62726-4 (PMC11143245; doi:10.1038/s41598-024-62726-4)
Supplement: Supplementary file 1 — Supplementary Information 1. [file 41598_2024_62726_MOESM1_ESM.docx]

**Efficacy and Safety of Stem Cell Transplantation for Multiple Sclerosis: A Systematic Review and Meta-analysis of Randomized Controlled Trials**

**Table S1.** Summary of the included studies.

| Study ID | Country | Study Design | Duration  , m | Stem cell therapy | | Comparator | Main Findings |
| --- | --- | --- | --- | --- | --- | --- | --- |
|  |  |  |  | **Intervention** | **Dose** |  |  |
| Li et al. 2014^35^ | China | Parallel RCT | 12 | Human umbilical cord derived MSCs + anti-inflammatory drugs | 4×10⁶ cells/kg once every 2 W for 3 consecutive times– IV infusion | Anti-inflammatory drugs | Both the EDSS scores and frequency of relapses were significantly lower in SCT arm than the control. By inflammatory cytokines analysis, stem cells-treated patients showed a shift from Th1 to Th2 immunity. No significant adverse events(AEs) occured during a year of follow-up. |
| Llufriu et al. 2014^34^ | Spain | Phase II, Cross-over RCT | 12^a^ | Autologous bone marrow MSCs + anti-inflammatory drugs | 1–2 ×10⁶ cells/Kg - IV infusion | Placebo + Anti-inflammatory | Mesemchymal stem cells (MSCs) were safe and didn't cause serious AEs. The change in radiological and clinical endpoints was nonsignificant. The decrease in Th1 (CD4+ IFN-c+) cells in blood of MSCs treated patients was nonsignificant. |
| Lublin et al. 2014^33^ | USA; Canada | Phase Ib, Parallel RCT | 12 | Mesenchymal-like cells from human placenta+ anti-inflammatory+DMTs | 150 x 10⁶ and 600 x 10⁶ cells (2 infusion for each arm) – IV infusion | Placebo | SCT caused the patients’ EDSS to be decreased or stable. No trends were seen in the MSFC, Fatigue, or Quality of Life scale. No patient experienced a dose-limiting toxicity. AEs were mild or moderate in severity. Serious infusion-related events occurred in 2 patients in the high-dose group. |
| Mancardi et al. 2015^38^ | Italy; Spain | Phase II, Parallel RCT | 48 | Autologous peripheral blood SCs +Immunosuppressive drugs | (3-8)x10⁶ cells/kg–IV infusion | Mitoxantrone + methylprednisolone | SCT significantly reduced MRI activity, the number of new T2 lesions were fewer in SCT arm than mitoxantrone through 4 years of follow-up. It also reduced GELs and the annualized relapse rate. No difference was found regarding the progression of disability. |
| Ferna´ndez et al. 2018^36^ | Spain | Phase I/II, Parallel RCT | 12 | Adipose-derived MSCs | 1 x 10⁶ and 4 x 10⁶ cells/kg– IV infusion | Placebo | AEs occurred were expected without treatment-related serious AEs. EDSS, MRI changes, and evoked potentials showed no significant differences. No significant changes occurred in CSF analysis, cognition, or quality of life. |
| Burt et al. 2019^37^ | US;UK; Sweden; Brazil | Parallel RCT | 12 | Autologous peripheral blood SCs+immunosuppressive drugs | 2 × 10⁶ cells/Kg – IV infusion | DMTs+ immunosuppressive drugs | SCT significantly improved patients’ EDSS more than DMT. MSFC, quality of life, and MRI T2 lesions volume significantly improved following SCT compared with the DMT group. There were no deaths or serious toxicities. |
| Petrou et al. 2020^32^ | Israel | Phase II, Cross-over RCT | 12 ^a^ | Autologous bone marrow MSCs | 1 × 10⁶ cells/Kg – IV infusion and intrathecal | Placebo | No serious or treatment-related AEs occurred and disability progression was significantly fewer in SCT arm. IV SCT was beneficial but less than the intrathecal route in terms of motor, cognitive, and radiological endpoints. |
| Uccelli et al. 2021^31^ | Austria;Canada;Denmark;France;Italy;Iran;Spain;Sweden;UK | Phase II, Cross-over RCT | 12 ^a^ | Autologous bone marrow MSCs | 1–2 × 10⁶ cells/ kg – IV infusion | Placebo | AEs were similar in both arms and serious AEs were not treatment-related. Compared with placebo, SCT didn’t significantly affect number of GELs, MRI lesions or clinical outcomes at 24 and 48 weeks. No carryover effect was detected. |
| Nabavi et al. 2023^39^ | Iran | phase I/IIa Cross-over RCT | 12 ^a^ | Autologous bone marrow MSCs | 2 × 10⁶ cells/kg – IV infusion | Placebo | The trial detected nonsignificant change in EDSS, MRI-T2 lesions, and CSF biomarkers analysis. No remarkable change was observed in patients’ cognitive functions and MRI GAD lesions. There was no treatment-related adverse events. |

Abbreviations: MSCs mesenchymal stem cells, IV intravenous, DMTs disease modifying therapies, SCT stem cell transplantation, EDSS expanded disability status scale, MSFC multiple sclerosis functional composite, MRI magnetic resonance imaging, GELs gadolinium-enhancing lesions.

^a^ Cross-over at 6 months.

**Table S2.** The change in efficacy endpoints reported after 6 months

| **Stuy ID** | **EDSS** | | **T25-FW** | | **9-HPT** | | **PASAT-3** | | **MRI lesions volume** | | **Number of relapses** | |
| --- | --- | --- | --- | --- | --- | --- | --- | --- | --- | --- | --- | --- |
|  | I | C | I | C | I | C | I | C | I | C | I | C |
| Li et al. 2014^35^ | -0.98(0.47) | 0.13(0.49) | - | - | - | - | - | - | - | - | - | - |
| Llufriu et al. 2014^34^ | 0.3(0.7) | 0.25(0.5) | 0.68(2.39) | -0.95(2.48) | -0.41(3.98) | 0.497(3.92) | - | - | 0.98(1.3) | 5.54(5.34) | 0.25(0.32) | 1.5(1.29) |
| Lublin et al. 2014^33^ | -0.57(1.43) | -0.37(0.48) | - | - | - | - | - | - | - | - | - | - |
| Mancardi et al. 2015^38^ | - | - | - | - | - | - | - | - | - | - | - | - |
| Ferna´ndez et al. 2018^36^ | - | - | - | - | - | - | - | - | - | - | - | - |
| Burt et al. 2019^37^ | -0.9(0.61) | 0.4(0.74) | -0.6(3.4) | 1.4(3.5) | -4.8(7.78) | 1.6(2.89) | 5.9(20.6) | 8.3(21.2) | -4.0(16.8) | 3.6(7) | - | - |
| Petrou et al. 2020^32^ | -0.06(0.51) | 0.63(0.89) | 2.71(16.69) | 1.77(5.47) | -1.61(3.22) | -1.41(5.39) | - | - | - | - | 0.19(0.47) | 0.44(0.63) |
| Uccelli et al. 2021^31^ | 0(0.76) | 0.17(0.38) | -0.3(1.67) | 0.13(0.98) | -0.17(3.4) | 0.43(2.65) | 2.67(6.06) | 3(5.3) | - | - | - | - |
| Nabavi et al. 2023^39^ | -0.25(0.83) | -0.67(0.69) | - | - | - | - | - | - | - | - | - | - |

-Data are presented as mean change (SD): mean change from baseline to 6 months and the standard deviation of mean change.

Abbreviations: I intervention arm (stem cell transplantation), C control, EDSS expanded disability status scale, T25-FW Timed 25 Foot Walk, 9-HPT Nine-hole peg test, PASAT-3 Paced auditory serial addition test, MRI magnetic resonance imaging .

**
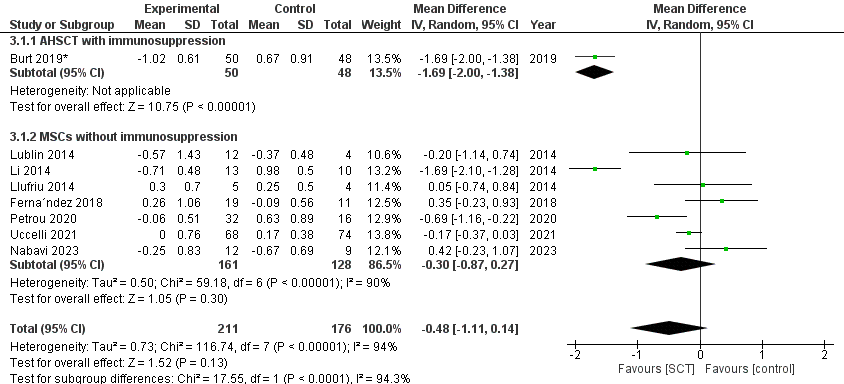
**

**Figure S1.** Forest plot of EDSS change from baseline.

*the study used immunosuppression before AHSCT


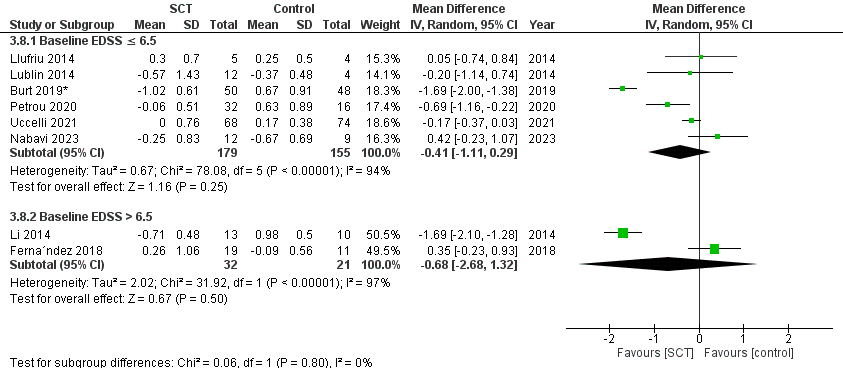


**Figure S2.** Forest plot of EDSS subgroup analysis according to patients’ baseline EDSS

*the study used immunosuppression before AHSCT


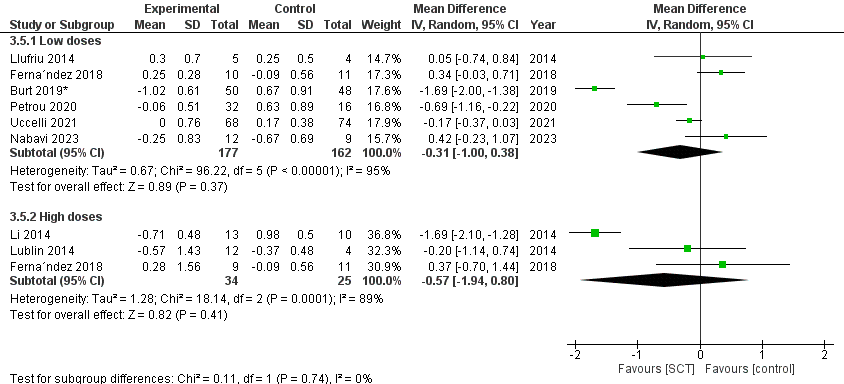
**Figure S3.** Forest plot of EDSS subgroup analysis according to the doses of transplanted stem cells

*the study used immunosuppression before AHSCT


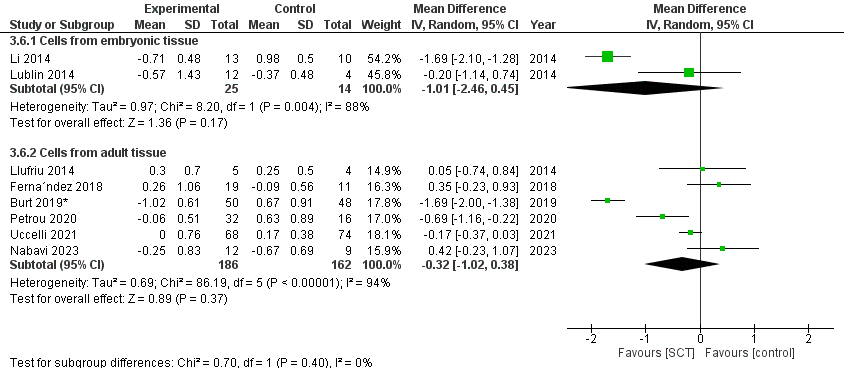


**Figure S4.** Forest plot of EDSS subgroup analysis according to the origin of stem cells

*the study used immunosuppression before AHSCT


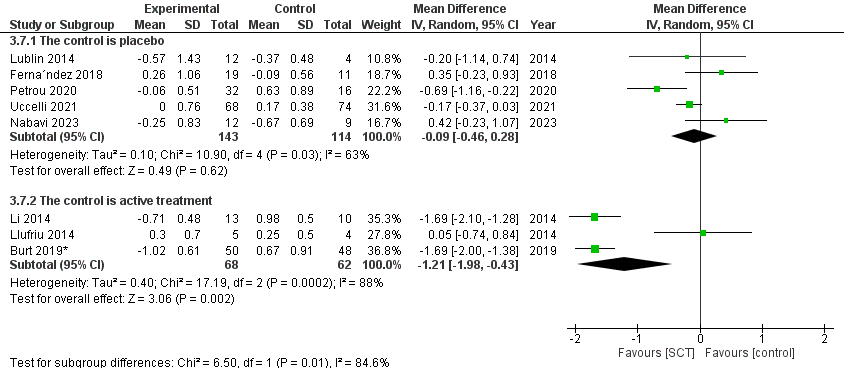
**Figure S5.** Forest plot of EDSS subgroup analysis according to the control group

*the study used immunosuppression before AHSCT


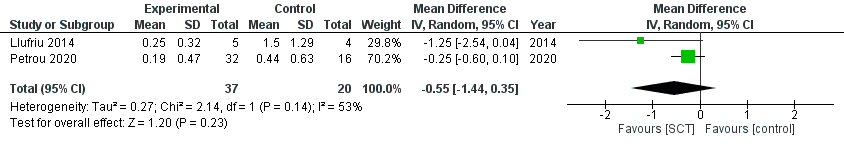


**Figure S6.** Forest plot showing relapses number during 6 months of follow up


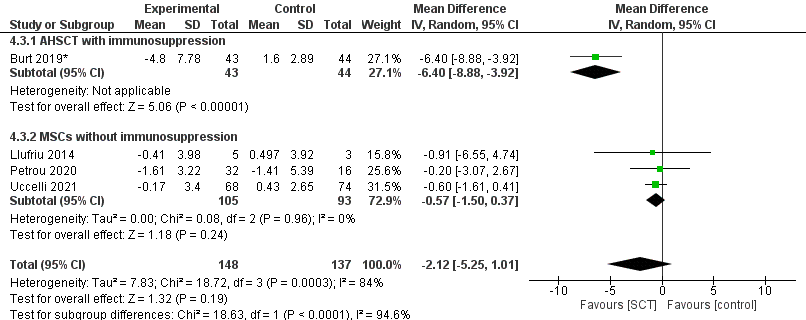


**Figure S7.** Forest plot of 9-HPT change from baseline

*the study used immunosuppression before AHSCT


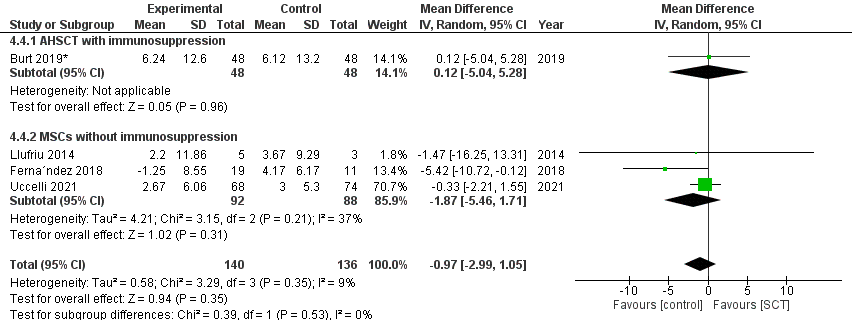


**Figure S8.** Forest plot of PASAT-3 score change from baseline

*the study used immunosuppression before AHSCT


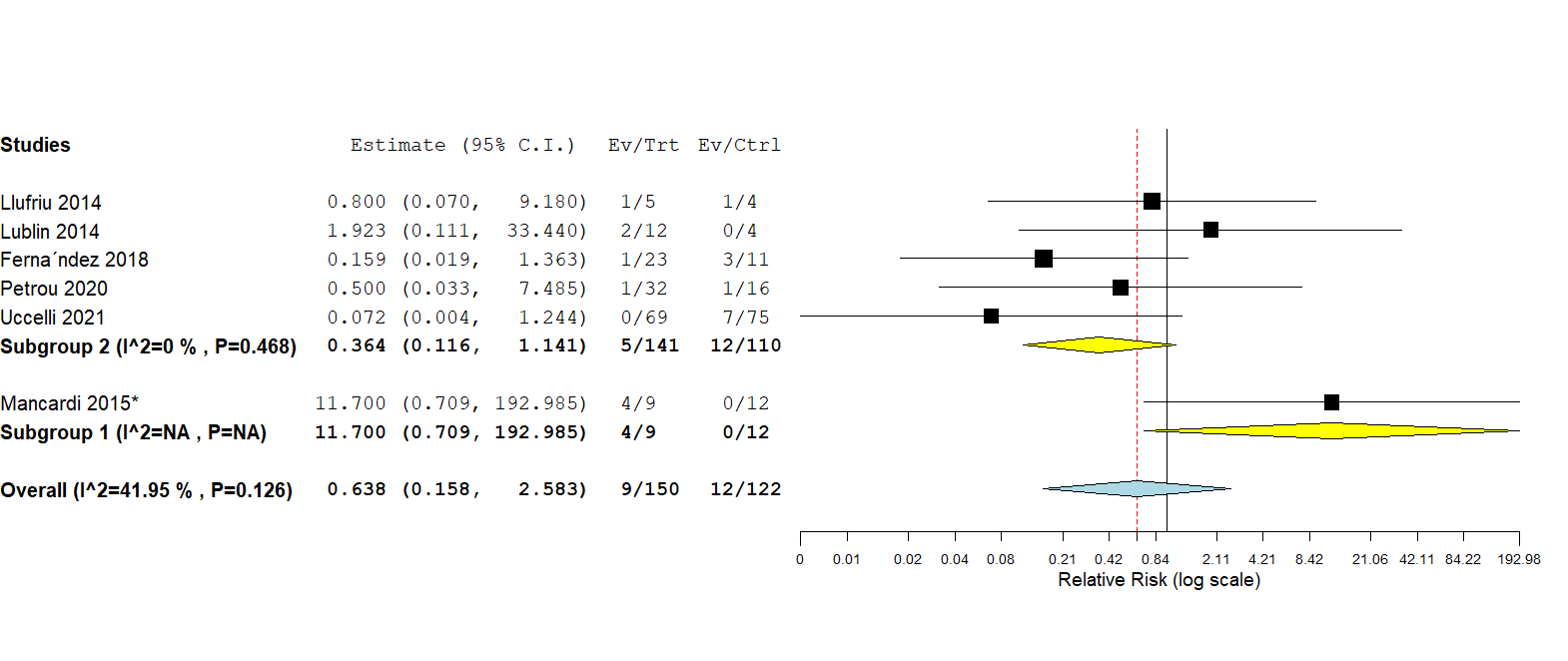
**Figure S9.** Forest plot showing incidence of serious adverse events

*the study used immunosuppression before AHSCT

Subgroup 1= AHSCT with immunosuppression


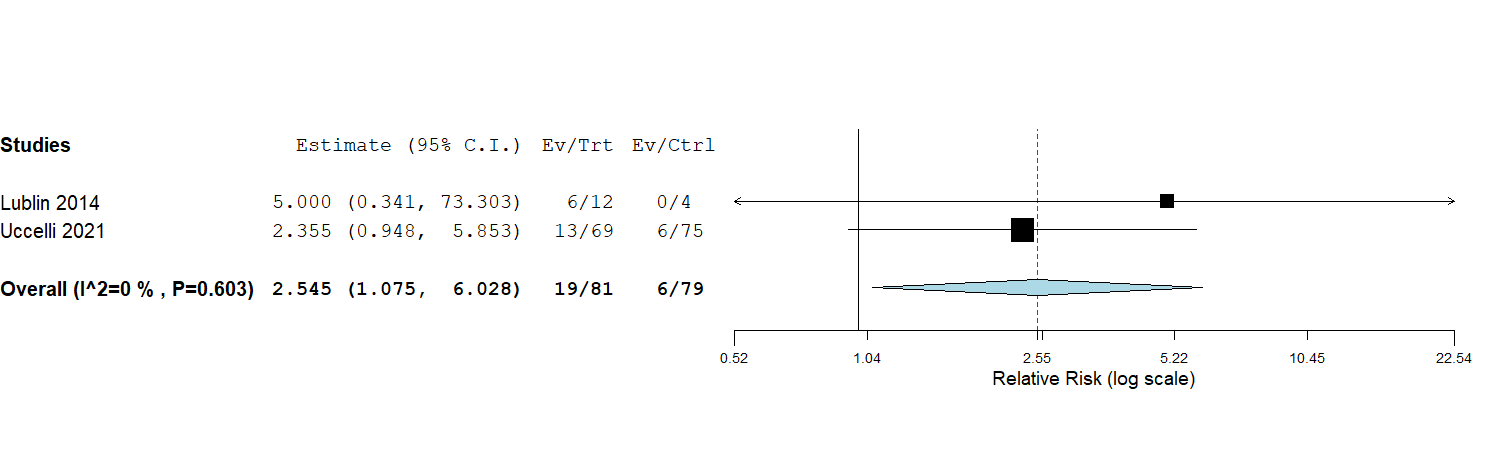
Subgroup 2= MSCs without immunosuppression

**Figure S10.** Forest plot showing incidence of administration site adverse events
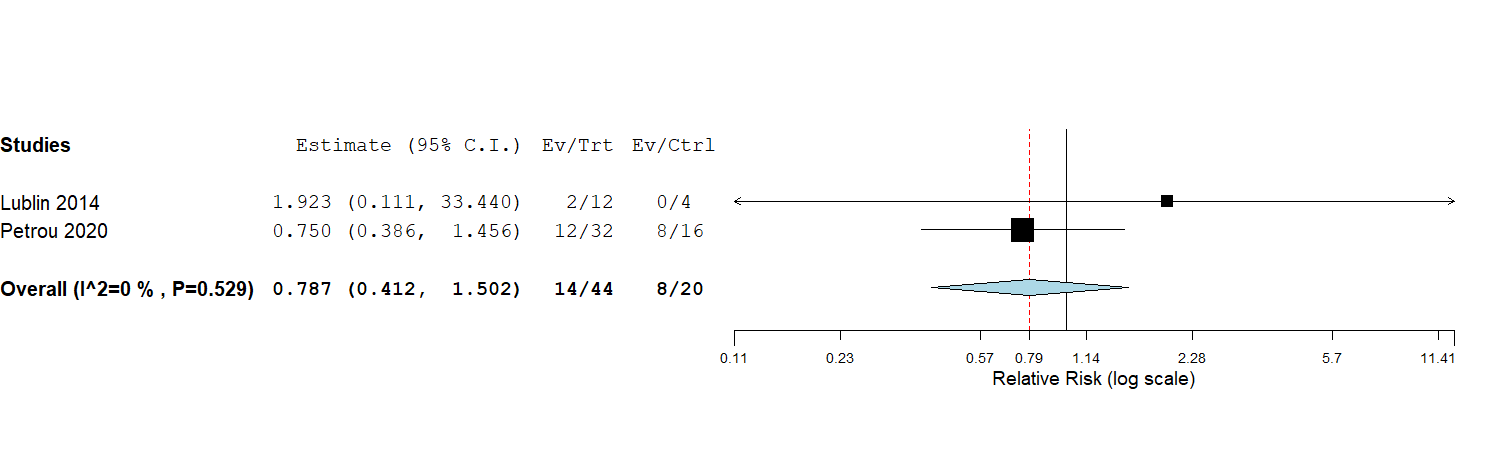
**Figure S11.** Forest plot showing incidence of headache


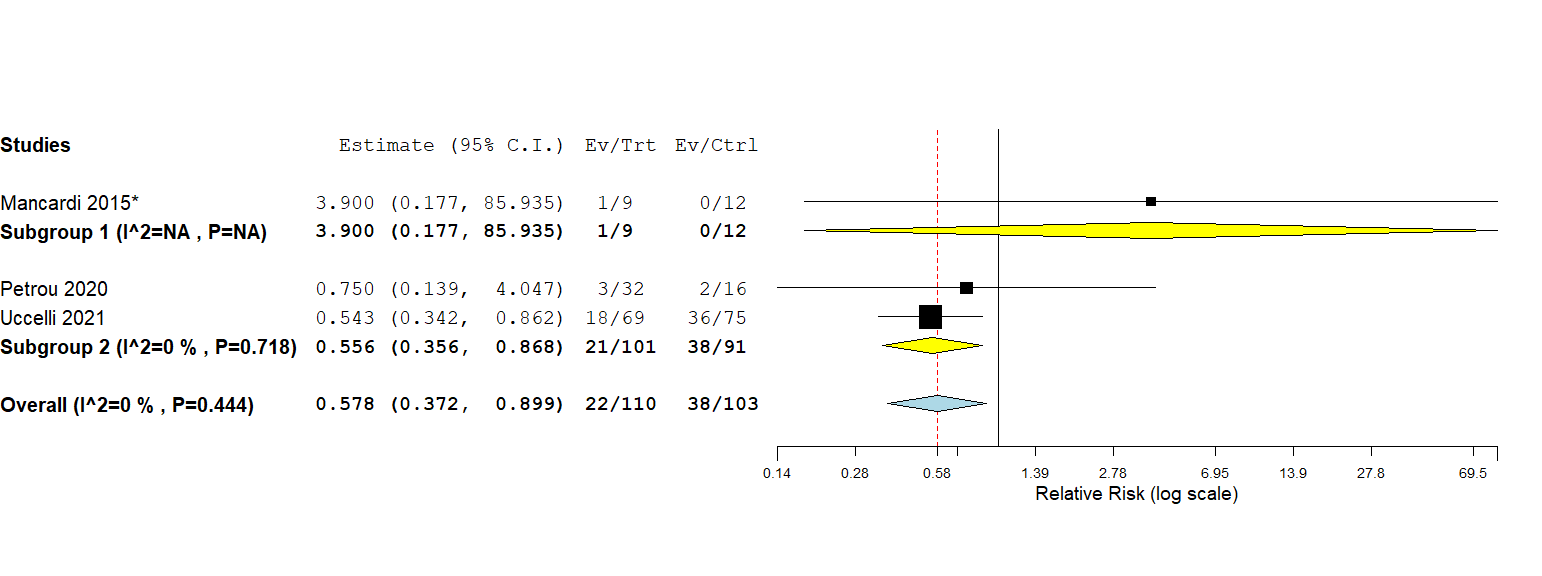


**Figure S12.** Forest plot showing incidence of total infections

*the study used immunosuppression before AHSCT

Subgroup 1= AHSCT with immunosuppression

Subgroup 2= MSCs without immunosuppression


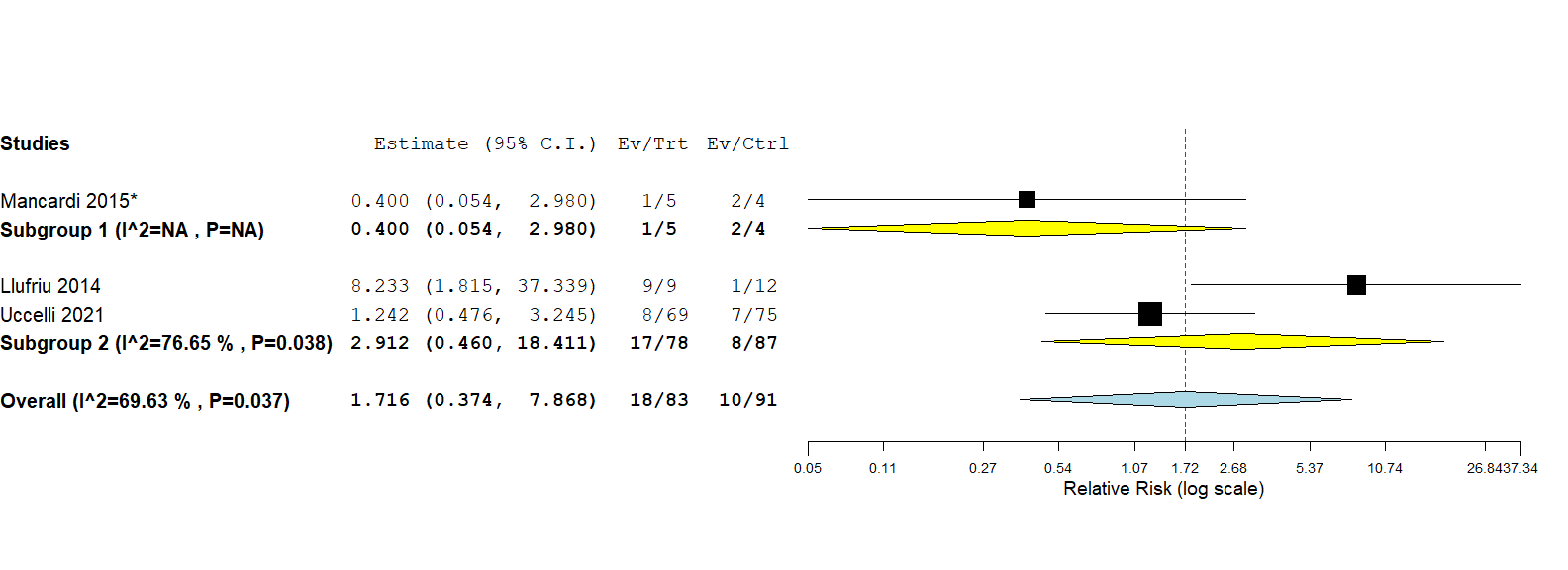
**Figure S13.** Forest plot showing incidence of gastrointestinal disorders

*the study used immunosuppression before AHSCT

Subgroup 1= AHSCT with immunosuppression

Subgroup 2= MSCs without immunosuppression

**
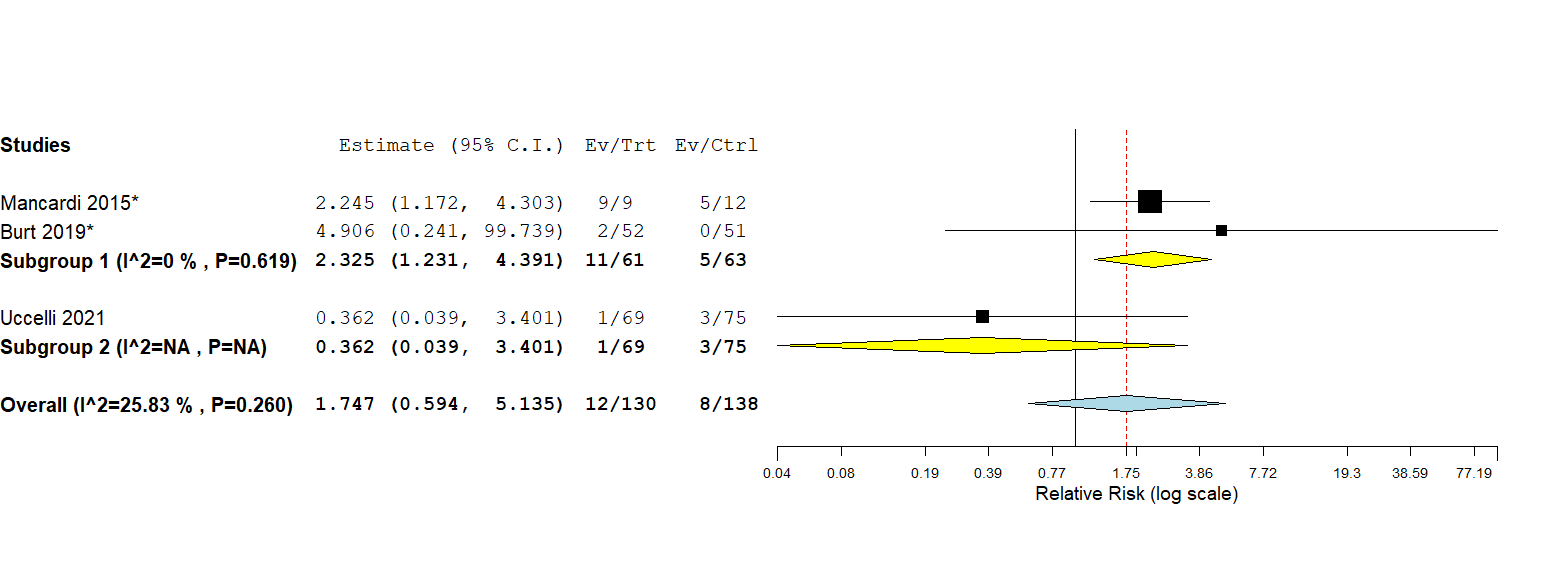
**

**Figure S14.** Forest plot showing incidence of blood and lymphatic system disorders

*the study used immunosuppression before AHSCT

Subgroup 1= AHSCT with immunosuppression

Subgroup 2= MSCs without immunosuppression


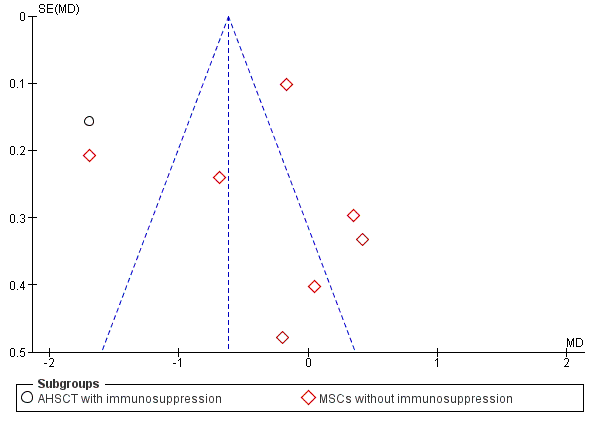


**Figure S15.** Funnel plot of EDSS change from baseline.
