# Supplementary material for: Efficacy and safety of stem cell transplantation for multiple sclerosis: a systematic review and meta-analysis of randomized controlled trials
Source: Sci Rep. 2024 May 31;14:12545. doi: 10.1038/s41598-024-62726-4 (PMC11143245; doi:10.1038/s41598-024-62726-4)
Supplement: Supplementary file 2 — Supplementary Information 2. [file 41598_2024_62726_MOESM2_ESM.docx]

**Efficacy and Safety of Stem Cell Transplantation for Multiple Sclerosis: A Systematic Review and Meta-analysis of Randomized Controlled Tria**
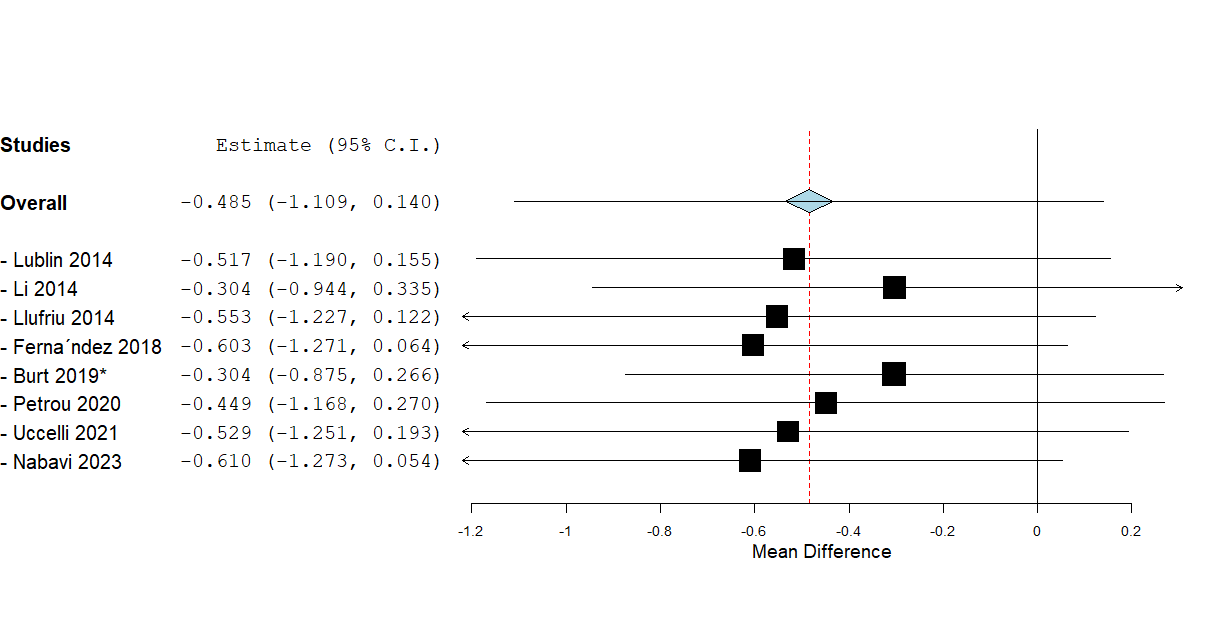
**Figure L1**: Leave-one out analysis of EDSS change from baseline.

*the study used immunosuppression before AHSCT


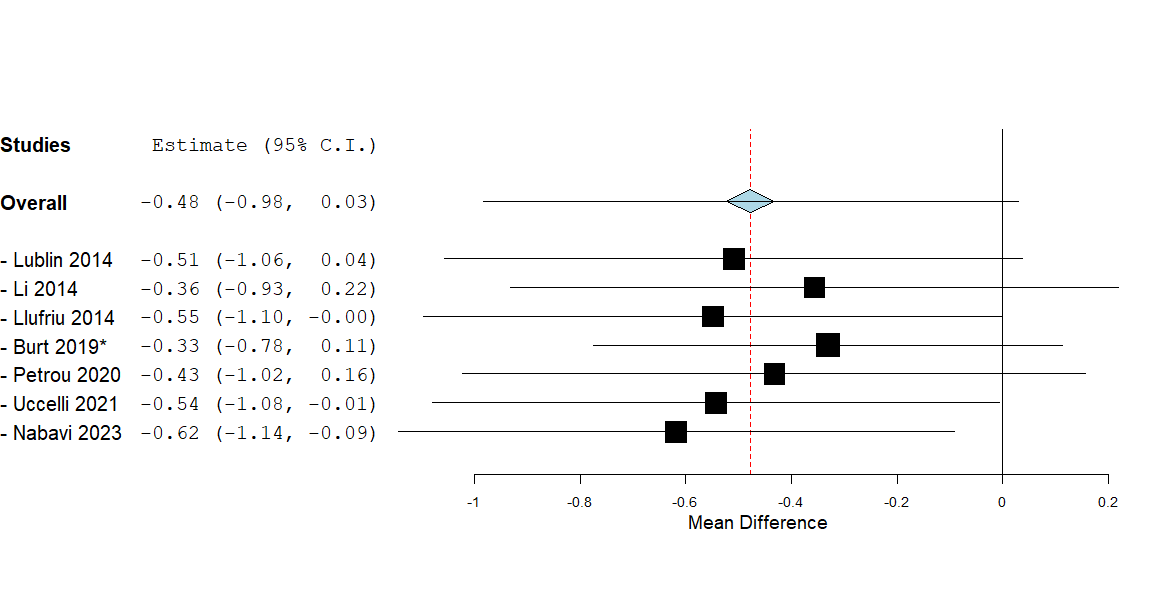
**Figure L2**: Leave-one out analysis of EDSS change at 6 months.

*the study used immunosuppression before AHSCT


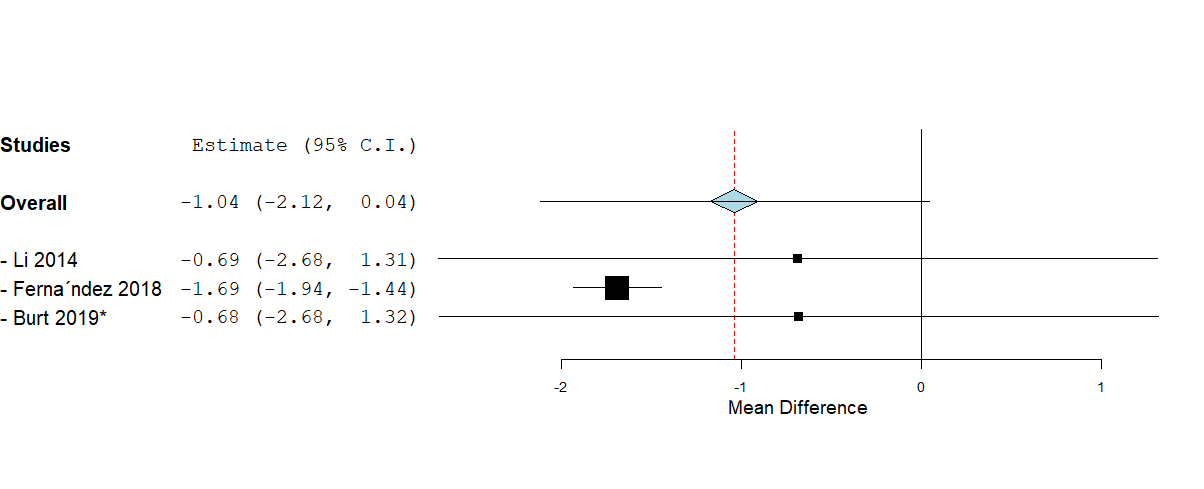


**Figure L3**: Leave-one out analysis of EDSS change at 12 months.

*the study used immunosuppression before AHSCT


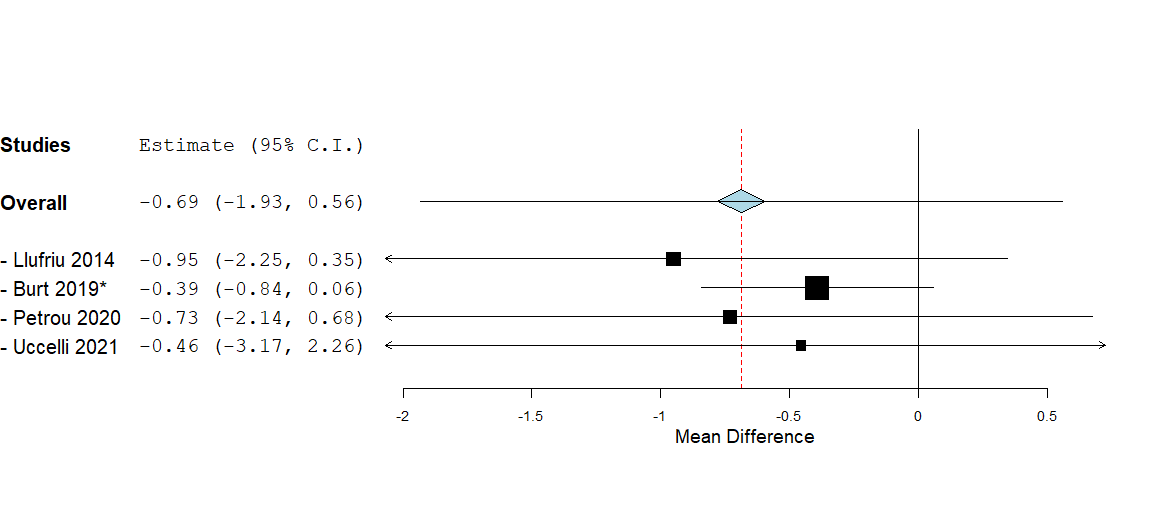


**Figure L4**: Leave-one out analysis of T25-FW change at 6 months.

*the study used immunosuppression before AHSCT


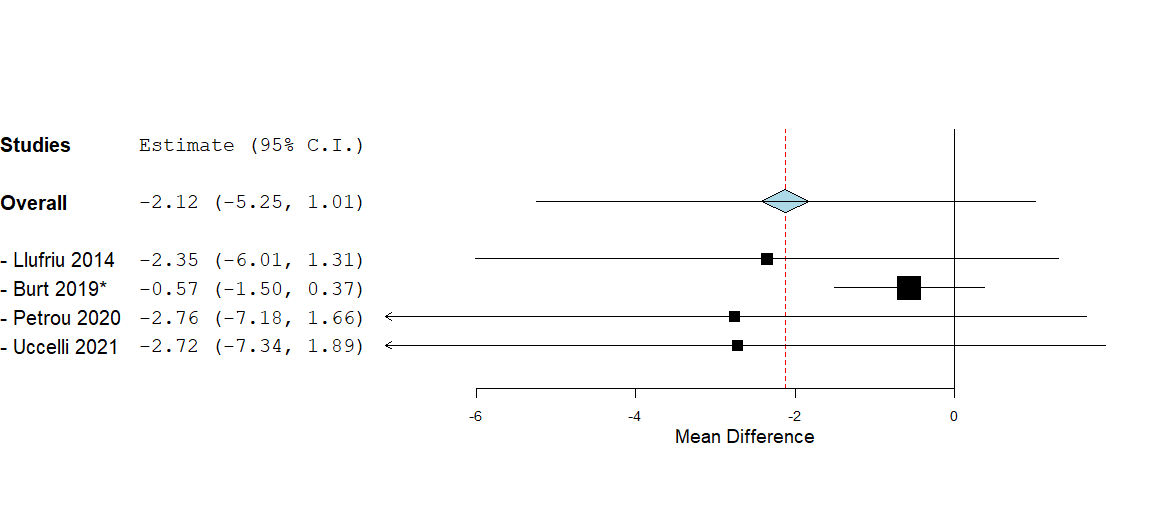
**Figure L5**: Leave-one out analysis of 9-HPT change at 6 months.

*the study used immunosuppression before AHSCT


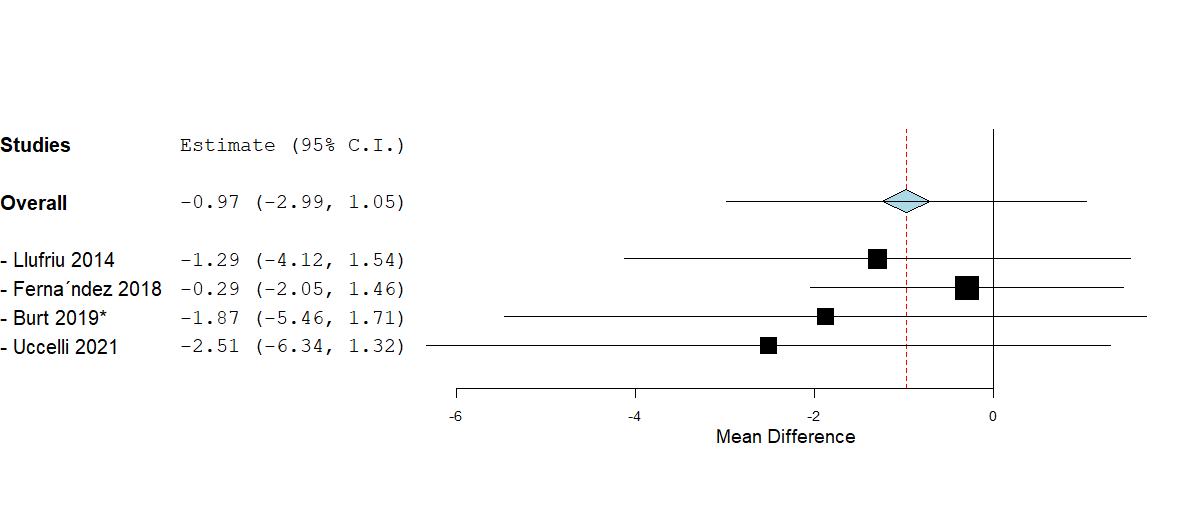


**Figure L6**: Leave-one out analysis of PASAT-3 score change.

*the study used immunosuppression before AHSCT


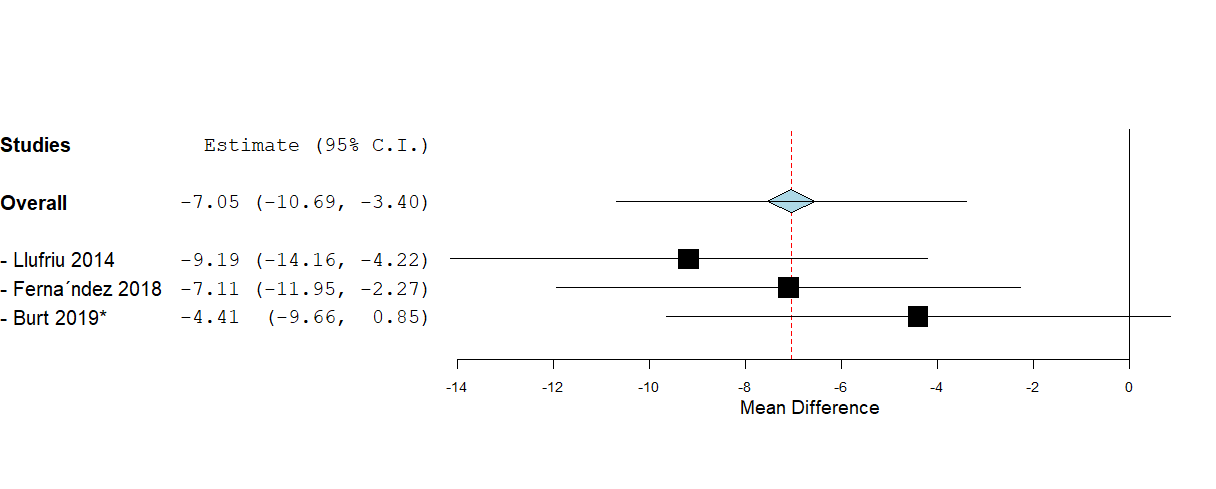
**Figure L7**: Leave-one out analysis of MRI lesion volume change.

*the study used immunosuppression before AHSCT


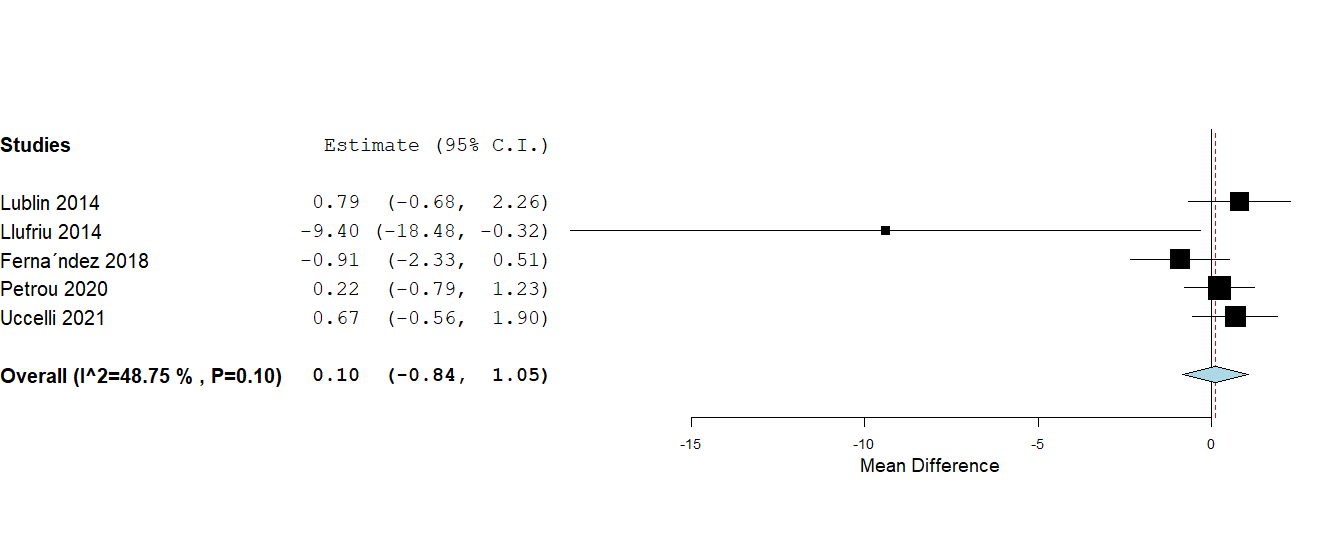


**Figure L8**: Leave-one out analysis of GELs number change.

**Table L1.** The significant results of the leave-one-out sensitivity analysis.

| **Outcome** | **Sensitivity analysis** | **No. of studies** | **No. of participants** | **Model** | **Effect of Estimate, MD, 95% CI** | **P-value** | **Heterogeneity, I^2^ (p-value)** |
| --- | --- | --- | --- | --- | --- | --- | --- |
| **EDSS (6 months)** | Before any exclusion. | 7 | 365 | Random | -0.48 [-0.98, 0.03] | 0.07 | 91% (p<0.00001) |
|  | After Nabavi et al. exclusion. | 6 | 344 | Random | -0.62 [-1.14, -0.09] | **0.02** | 91% (p<0.00001) |
| **EDSS (12 months)** | Before any exclusion. | 3 | 151 | Random | -1.04 [-2.12, 0.04] | 0.06 | 95% (p<0.00001) |
|  | After Ferna´ndez et al. exclusion. | 2 | 121 | Random | -1.69 [-1.94, -1.44] | **<0.00001** | 0% (p=1) |
| **MRI T2 lesion volume change** | Before any exclusion. | 3 | 136 | Random | -7.05 [-10.69, -3.40] | **0.0002** | 0% (p=0.38) |
|  | After Burt et al. exclusion. | 2 | 39 | Random | -4.41 [-9.66, 0.85] | 0.1 | 0% (p=0.77) |

Abbreviations: MD mean difference, CI confidence interval, EDSS expanded disability status scale, MRI magnetic resonance imaging .
